# Dimensional adversity, brain-age, & mental health: Differences in male and female adolescents

**DOI:** 10.1016/j.dcn.2026.101671

**Published:** 2026-01-13

**Authors:** Michelle Shaul, Sarah Whittle, Niousha Dehestani, Timothy J. Silk, Nandita Vijayakumar

**Affiliations:** a School of Psychology, Faculty of Health, Deakin University, Burwood, Victoria, Australia; b Centre for Youth Mental Health, The University of Melbourne, Victoria, Australia; c Orygen, Parkville, Victoria, Australia; d Developmental Imaging, Murdoch Children’s Research Institute, Parkville, Victoria, Australia; e Centre for Adolescent Health, Murdoch Children’s Research Institute, Parkville, Victoria, Australia

**Keywords:** Adolescent Brain Cognitive Development (ABCD) Study, Early life adversity, Threat, Deprivation, Unpredictability, Socioeconomic status, Sex differences, Mediation, Childhood, Adolescence, Mental Health, Brain development

## Abstract

Early life adversity (ELA) has been linked to shifts in developmental pace. This study examined whether brain maturity during early adolescence was influenced by ELA, and whether it explained the relationship between ELA and mental health problems. A sample (*n* = 7658, 46 % female) from the Adolescent Brain and Cognitive Development (ABCD) Study was utilized, with data collected at three time points spanning 9–14 years of age. Exposure to threat, psychosocial deprivation, household instability, and socioeconomic stress were measured at baseline. A predictive model of normative brain development (brain age) trained on a large independent lifespan sample was applied to structural neuroimaging data from the second timepoint. Brain-age-gap (BAG) – the difference between model predicted brain age and chronological age – was tested as a mediator of adversity exposure and internalizing/externalizing problems at the third timepoint. A more positive BAG was associated with more externalizing problems, but hypothesized associations between adversity and BAG were not significant. Sex moderation of these pathways suggests adversity may differentially affect the pace of brain development for males and females, which uniquely explains vulnerability to externalizing problems. The findings highlight the importance of examining sex-specific effects of adversity on adolescent development and mental health.

## Highlights

• Bayesian analysis tested brain-age-gap mediation in longitudinal ABCD Study.
• Dimensional adversity model: threat, deprivation, unpredictability & SES.
• Sex moderated dimensions of early life adversity with brain & psychopathology.
• Males: psychosocial deprivation predicted delayed structural brain maturation.
• Females: older brain age predicted adolescent externalizing problems.

## 1 Introduction

Early life adversity (ELA) has been consistently linked to later mental health problems (Bellis et al., 2019) and a growing literature points to differences in biological ageing across multiple body systems in ELA-exposed individuals (Lyons et al., 2023). Developmental pacing of neural maturation during adolescence represents a potential mechanism linking adversity to subsequent psychopathology (Gee, 2021, McLaughlin et al., 2020). Adolescence is marked by significant neural morphometric changes, including normative synaptic pruning and refinement during the transition into adulthood (Konrad et al., 2013). However, determining whether phenotypic differences in the brain following ELA represent shifts in developmental pacing has been challenging, given that normative adolescent neural development follows non-uniform timing and direction (e.g., linear or non-linear) of maturation depending on the region and morphometric parameter (e.g., cortical thickness, surface area and volume) examined (Bethlehem et al., 2022, Vijayakumar et al., 2016).

Recent availability of large open science neuroimaging datasets and the application of machine learning models provide new opportunities for understanding the relationship between environmental factors, neural maturation, and mental health outcomes. Machine learning models trained on large neuroimaging datasets learn patterns of age-related variation in brain morphometry to predict an individual’s “brain age” from magnetic resonance imaging (MRI) data (Cole and Franke, 2017). This predicted brain age reduces high dimensional neuroimaging data into a single summary metric, though the specific features incorporated vary depending on the imaging modality used in model training. Brain-age-gap (BAG) is calculated as the difference between an individual’s predicted brain age and their chronological age. BAG provides an index of how an individual’s brain morphometry compares to an age norm (Franke and Gaser, 2019, Truelove-Hill et al., 2020). Typically, positive BAG values are interpreted as indicative of an older appearing brain, while negative values are interpreted as a younger appearing brain. Investigations are ongoing into the longitudinal stability of BAG across childhood and adolescence (for discussion see Whitmore and Beck, 2025). However, preliminary evidence links BAG with broad adolescent mental health outcomes, with some heterogeneity in the direction of effects that may relate to methodological (e.g., model and neuroimaging parameters) and sample features (e.g., age and sex distribution). Specifically, positive BAG has been associated with greater general psychopathology, psychosis/psychotic disorders as well as obsessive-compulsive and internalizing symptoms (Chung et al., 2018, Cropley et al., 2021, Drobinin et al., 2022, MacSweeney et al., 2025, Truelove-Hill et al., 2020). Meanwhile, general psychopathology (Luna et al., 2021, Lund et al., 2022) and internalizing symptoms (Cohen et al., 2024) have also been associated with negative BAG, along with generalized anxiety (Zhou et al., 2023). Despite this heterogeneity, BAG may provide an appropriate marker of individual variability in normative neural development to test as a putative pathway from ELA to psychopathology.

Dimensional models of adversity provide a framework for examining these relationships that may be more informative than cumulative adversity scores or examination of singular adversities (for discussion see McLaughlin et al., 2021). The dimensional model of adversity and psychopathology (DMAP; McLaughlin et al., 2014) and evolutionary-developmental model (Ellis et al., 2009) conceptualize ELA along dimensions based on the underlying characteristics of threat, deprivation, and environmental unpredictability (Ellis et al., 2022). Threat-related adversities comprise experiences of serious risk of injury to self or others through intention (e.g., abuse or family violence) or accident/illness. Deprivation-related adversities are characterised by a diminished availability of evolutionarily expectant stimuli, such as neglect of bioenergetic (e.g., food insecurity) or psychosocial (e.g., lack of cognitive and social stimulation) needs. Unpredictability refers to the degree of stochastic variability in environmental harshness (i.e., consistency of the adversity exposure) as well as the degree of predictability in the availability and responsiveness of caregivers (Young et al., 2020). These characteristics are thought to differentially affect development and vulnerability to psychopathology (Chahal et al., 2022, Kasparek et al., 2023, McLaughlin and Sheridan, 2016, Miller et al., 2018, Schafer et al., 2022, Usacheva et al., 2022). Notably, although some studies have examined socioeconomic status as a measure of material and cognitive deprivation, socioeconomic status could be considered a complex exposure involving both deprivation and threat (Murgueitio et al., 2024, Vogel et al., 2021). Accordingly, in this paper, we distinguish between socioeconomic stressors (SES) and deprivation.

The few extant studies examining associations of ELA and brain age show mixed findings. In adolescent samples, the most consistent finding is an association between older appearing neural phenotypes with threat-related adversities (Beck et al., 2025, Drobinin et al., 2022, Gur et al., 2019) and low socioeconomic status (Beck et al., 2025, Cohen et al., 2024, Gur et al., 2019, Rakesh et al., 2021). However, inconsistencies exist. For example, Keding et al. (2021) found that deprivation (physical neglect), but not threat exposure, was associated with positive BAG in 8- to 18-year-old females. Yet deprivation measured as emotional neglect has been associated with a negative BAG – or a younger appearing neural phenotype (Beck et al., 2025). Furthermore, early life markers of unpredictability, such as parental mental illness or substance use and separation from biological parents, have been associated with older appearing brains (Beck et al., 2025, Cohen et al., 2024). Despite some heterogeneity, findings generally align with theoretical predictions from dimensional adversity models. However, whether these adversity-related shifts are a putative pathway to psychopathology remains untested. Furthermore, existing studies have not considered sex differences despite evidence for sex-specific neural correlates of ELA (Bath, 2020, Donovan et al., 2024, Helpman et al., 2017, Hodes and Epperson, 2019, White and Kaffman, 2019).

The current study sought to address these limitations using data from the Adolescent Brain Cognitive Development (ABCD) Study, a longitudinal cohort study of adolescents from the United States. We examine BAG as a potential mediator between four adversity dimensions (threat, psychosocial deprivation, household instability, and SES) and transdiagnostic mental health outcomes (internalizing and externalizing problems). Based on our previous findings with a similar sample (Shaul et al., 2024), we expected positive associations between the four adversity dimensions and both mental health problem scales. We hypothesized that greater threat exposure would be associated with a positive BAG, and that this would mediate the association between threat exposure and mental health problems, particularly internalizing problems. Given the limited research and inconsistent findings on other adversity dimensions, we made no specific directional hypotheses for deprivation, SES, or household instability. As an exploratory aim, we examined sex differences in the associations between ELA, BAG, and mental health problems.

## 2 Methods

### 2.1 Participants

Data was drawn from the ongoing longitudinal ABCD study, releases 4.0 and 5.1 (accessed via the National Institute of Mental Health Data Archive’s ABCD Collection). The ABCD sample is a community cohort of over 11,800 youth recruited from 21 sites across the US (Garavan et al., 2018). Data for this study was taken from three timepoints: baseline, two-year follow-up, and three-year follow-up, spanning ages 9- to 14-years old. A flow chart of participant inclusion is presented in Figure S1. Participants with available (minimally processed) T1-weighted MRI data at two-year follow-up that met ABCD’s quality criteria were eligible for inclusion (*n* = 7693). Of these participants, those with missing data on all variables of interest or missing identifier or key demographic (i.e., race/ethnicity) data were excluded. A further 32 participants were excluded following manual quality control checks on T1-weighted data resulting in a final analytic sample of *n* = 7658. All other missing data was imputed (described below).

### 2.2 Measures

#### 2.2.1 Dimensional adversity exposure

Dimensional adversity exposure was operationalised as latent factor scores from a confirmatory factor analysis (CFA), developed in our previous study of adversity in the ABCD cohort (Shaul et al., 2024). An overview of the questionnaires and items used are available in Tables S1 and S2. Sixty-eight questionnaire items from baseline were submitted to a theory-driven CFA. Items were allocated to one of four factors: threat, deprivation, SES or household instability. A 50–50 random hold-out method was used for model development. Adequate model fit was assessed as root mean square error of approximation (RMSEA) of ≤ .05, and comparative fit index (CFI) of ≥ .95 (Hu and Bentler, 1998). Further details on model development are available in Shaul et al. (2024). In the final model, the *threat* factor included items relating to experiencing or witnessing interpersonal violence/abuse/bullying and damage or injury from accidents. The *deprivation* factor included measures of psychosocial neglect, relating to family cohesion, caregiver emotional availability and caregiver supervision. The *SES* factor included measures of financial hardship (i.e., income-to-needs ratio, mean parent education) and neighborhood disadvantage (i.e., area deprivation index). The *household instability* factor included items relating to the consistency of caregivers, such as whether the child lives with non-biological parents (e.g., is the child adopted, in custodial care) and caregiver mental illness and substance use.

This pre-established model was applied to the current study sample and latent factor scores of the four adversity dimensions were extracted for each participant. These scores provide a continuous measure of the individuals exposure to each adversity dimension based on the weighted contribution of multiple ELA exposures at baseline, with higher scores indicating higher adversity exposure (i.e., lower scores for the SES factor indicate higher socioeconomic status/less adversity). All adversity dimensions were z-score normalized.

#### 2.2.2 Brain-age-gap

Participants’ brain-predicted age was calculated based on minimally processed T1-weighted images from the ABCD dataset at two-year follow-up (Hagler et al., 2019). These were acquired with real time motion correction and parameter harmonization across three 3 T scanner platforms used in the ABCD protocol, as described in full by Casey et al. (2018). A publicly available regression-based simple fully convolutional network (SFCN-reg) machine learning model by Leonardsen et al. (2022) was used to estimate participants’ brain age. This model has been trained and validated on minimally processed T1-weighted MRI data from *n* = 53542 individuals (52 % female) aged between 3 and 95 years old. It has achieved good predictive accuracy on the testing dataset (mean absolute error [MAE] of 2.47) and an external dataset (MAE = 3.90) employing different scanners (Leonardsen et al., 2022).

We applied skull-stripping and spatial normalization (using FreeSurfer v5.3) to the minimally processed T1-weighted images in ABCD, for consistency with Leonardsen et al. (2022). Following the standardized procedure, a full age-bias correction was applied to brain age predictions, which addresses the systematic over- and underestimation of age at the extremes of the age distribution. Predicted age was deconfounded for chronological age during model training using a joint regression approach, and the learned correction parameters were subsequently applied to the test data, producing age-bias-free estimates (Leonardsen et al., 2022). Predictive accuracy was assessed by estimating the proportion of variance in observed age explained by predicted age (*r*^2^) and the mean absolute error (MAE).

Each participants’ brain-predicted age was subtracted from their chronological age at the two-year follow-up. A resultant BAG of zero indicates brain development that is consistent with the normative model and thus might be considered “on time”. Negative values indicate a predicted age less than chronological age, suggesting a younger appearing brain compared to the normative model, while positive values suggest an older appearing brain.

#### 2.2.3 Mental health problems

Caregiver reported raw summed scores from the Child Behavior Checklist (CBCL) for Ages 6–18 (Achenbach, 2009) for internalizing and externalizing problem scales at three-year follow up were used as outcome measures. Respondents were asked to rate the presence of behaviours and emotional states over the past 6 months on a 3-point scale from 0 (*not true*), 1 (*somewhat true*), or 2 (*very true*).

#### 2.2.4 Covariates

Several covariates were added to the model to account for potential confounding effects. As participants were nested within data collection sites and family units (sibling pairs), these group-level identification variables were included. Additionally, as the collection date for some participants’ three-year follow up was prior to the COVID-19 pandemic, a binary covariate was included to indicate whether collection was before or after the start of the pandemic on March 11, 2020 (World Health Organisation). Demographic variables of sex assigned at birth (*Female* and *Male*) and race/ethnicity (*non-Hispanic White*, *non-Hispanic Black*, *non-Hispanic Asian, Hispanic*, and *Other*) were included in all models. These were categorical variables based on caregiver report. The *Other* race/ethnicity category included those who identified as multiracial/multiethnic, Native American/Alaskan Native, Native Hawaiian or Other Pacific Islander. Race/ethnicity was included as a proxy variable to account for the additional adversity experienced by minority racial and ethnic groups (Shonkoff et al., 2021) as well as the observed differences in the occurrence of mental health problems (Lopez et al., 2017).

### 2.3 Statistical analyses

*RStudio* (RStudio Team, 2020) was used for all data processing and analyses. Please see supplementary materials for details on packages, code, model specifications, and equations.

#### 2.3.1 Imputation

Imputation was conducted on the full sample (*n* = 11865) prior to application of exclusion criteria to maximise the amount of predictive data for imputation. Information on missingness is presented in Table S3. Across variables of interest (baseline adversity variables and internalizing and externalizing problems), 2.81 % of the data was missing. Missing values were estimated as an average of imputed datasets using fully conditional specification (detailed in Supplementary Materials). The decision to use averaged values in analysis was made to reduce computational demands, which would have been onerous given the complexity and quantity of analyses. Although it is acknowledged that this approach is a limitation, as the resulting estimates cannot account for between-imputation variability (Van Buuren, 2012).

#### 2.3.2 Mediation models

**Fig. 1:**
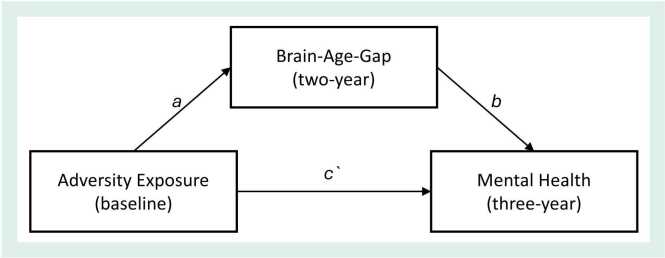
*Proposed Mediation Model. Note*. Proposed indirect model wherein associations between dimensional adversity exposure measured at baseline (threat, deprivation, socioeconomic stress, and household instability) and internalizing and externalizing problems at three-year follow-up are mediated by BAG estimated using T1-weighted MRI scans at two-year follow-up.

The proposed mediation model is outlined in Fig. 1. Bayesian regression was used to examine potential direct and indirect effects of adversity exposure on mental health problems via BAG. Mixed-effects multivariate models were run separately for each pairing of the adversity predictors (threat, deprivation, SES, instability) and mental health outcomes (internalizing, externalizing), with BAG as the proposed mediator (eight models in total). The following regression equations were simultaneously calculated for each model:

i. BAG ∼ adversity dimension + covariates
ii. mental health ∼ BAG + adversity dimension + covariates

The covariates in each equation were sex, race/ethnicity, and COVID-19 as fixed level-one variables and, to account for the nested data, family and site IDs as level-two variables with random intercepts.

Weakly informative Student’s *t* priors (μ = 0, σ = 1, v = 3) were specified for the population-level (fixed effect) parameters and *brms* default priors were deemed appropriate for remaining parameters (Vehtari, 2024) given the large sample size (Gelman et al., 2017). Equation *i* was assessed using a skew normal distribution as BAG had a slight positive skew (Figure S2), while equation *ii* was assessed using a Poisson distribution as values of the mental health variables were positively skewed and could not fall below zero.

A product of coefficients approach was used to test mediation with methods outlined by Yuan and MacKinnon (2009). Specifically, the indirect (mediation) effect was calculated as the product of the regression coefficients of the *a* and *b* paths (using the posterior samples from Bayesian regression models to estimate a distribution). For all paths of interest, the mean and 95 % quantile intervals of the posterior distributions were extracted as effect estimates and credible intervals (CIs). Bayesian CIs represent the range within which the effect estimate had 95 % probability of falling within based on the posterior distribution. Effects were considered present when the CIs did not include zero. To assess the robustness of findings, 99 % CIs were also calculated. Furthermore, two sets of sensitivity analyses examined whether effects remained when i) controlling for baseline internalizing and externalizing problems and ii) co-occurring adversity exposure.

#### 2.3.3 Exploratory analyses: sex differences

Potential sex-moderating effects on the mediation models were examined. The eight models above were re-run with the inclusion of a sex interaction on the predictor variables:

i. BAG ∼ adversity dimension*sex + covariates
ii. mental health ∼ BAG*sex + adversity dimension*sex + covariates

Models were tested for moderated mediation (Figure S5) using the product of coefficients method outlined by Hayes and Rockwood (2019). Specifically, whether in the above mediation model (Fig. 1), sex moderated the *a* path (first-stage moderated mediation) or the *b* path (second-stage moderation). Moderating effects were considered present when the 95 % CIs did not include zero. When moderation was identified, mediation models were re-run separately for males and females as post-hoc analysis to further understand the effects.

## 3 Results

Characteristics of the final sample are provided in Table 1. Participants excluded from the analytic sample were more likely to identify as of non-Hispanic Asian or Hispanic origin, be assigned male at birth, and be slightly older (∼3 months; Table S5, Figure S6). Correlations between variables of interest are presented in Table S6.

**Table 1:** Characteristics of Final Analytic Sample.

		Females			Males	
		*Freq/M(SD)*	*%/Min-Max*		*Freq/M(SD)*	*%/Min-Max*
	*n*	3547	46 %		4111	54 %
Age (Months)						
	*Baseline*	118 (7.40)	107–132		119 (7.50)	107–133
	*Two-Year*	143 (7.80)	128–166		144 (7.70)	127–164
	*Three-Year*	154 (7.60)	137–177		155 (7.70)	137–173
Adversity Factors (Baseline)						
	*Threat*	-0.003 (0.99)	-1.90–4.90		0.004 (1.00)	-1.90–5.10
	*Deprivation*	-0.10 (1.00)	-2.10–3.40		0.09 (0.98)	-2.10–3.90
	*SES*	0.02 (1.00)	-3.90–1.80		-0.02 (1.00)	-3.90–1.80
	*Instability*	0.01 (1.00)	-1.80–4.30		-0.01 (0.99)	-1.80–3.70
Brain Age Gap (Two Year)		0.05 (1.00)	-2.10–5.60		-0.04 (0.97)	-2.10–5.00
Mental Health (Three Year)						
	*Internalizing Problems*	5.80 (6.30)	0–40		4.80 (5.50)	0–44
	*Externalizing Problems*	3.60 (5.00)	0–41		4.60 (6.10)	0–48
Area Deprivation Index		41 (26)	1–100		39 (26)	1–100
Race/Ethnicity						
	*NH-White*	1893	53 %		2328	57 %
	*NH-Black*	698	20 %		781	19 %
	*Hispanic*	493	14 %		517	13 %
	*NH-Asian*	392	11 %		412	10 %
	*Other*	71	2 %		73	2 %
COVID-19						
	*During*	2972	84 %		3438	84 %
	*Prior*	575	16 %		673	16 %

*Note.* Freq, frequency; M, mean; SD, standard deviation; %, percentage of sex-specific sample; Min, minimum value; Max, maximum value; *n,* number of participants; NH, non-Hispanic; COVID-19, indicates whether three-year follow-up data was collected during or before the March 2020 pandemic.

### 3.1 Confirmatory factor analysis

The pre-established four factor model had adequate fit in the current sample: χ^2^(1111, *n* = 11865) = 17693.70, *p* < .001, RMSEA [90 % CI] = .035 [.035,.036], CFI = .970. Item loadings for this model are presented in Table S7 and S8.

### 3.2 Brain-age-gap

**Fig. 2:**
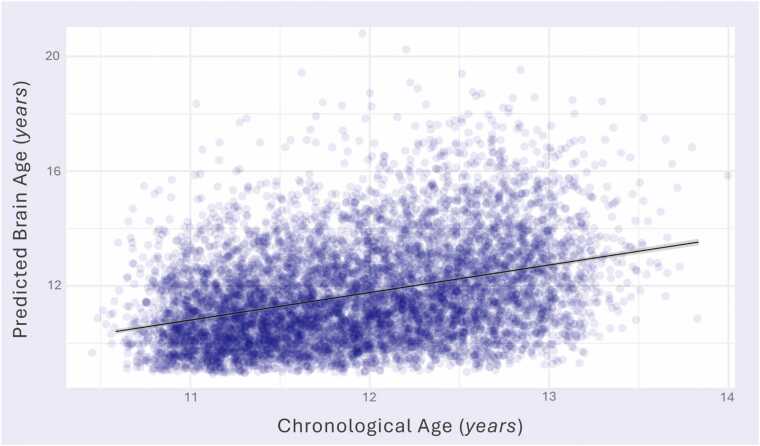
*Relationship between Brain Age and Chronological Age. Note.* Scatterplot depicting the relationship between chronological age (years) at 2-year follow up with participants’ predicted age (years) based on T1-weighted MRI images at 2-year follow up. Linear regression line shown in black.

Fig. 2 shows the association between predicted brain-age and chronological age (*R*^*2*^ =.13, *F*(1,7656) = 1099, *p* < .001). This correlation is slightly smaller than some previous studies using ABCD Study T1-weighted imaging (Dehestani et al., 2023, MacSweeney et al., 2025), which is likely due to the age window of the prediction model being much wider than the testing data. The model predicted age with a mean absolute error of 1.33, which is consistent with previous studies using this prediction model on the ABCD dataset (Holm et al., 2023) and previous adolescent BAG studies (Franke and Gaser, 2019)*.* The distributions of BAG for males (*M* = −0.30, *SD* = 1.60, min = −3.70, max = 8.00]) and females (*M* = −0.15, *SD* = 1.70, min = −3.70, max = 8.90) are presented in Figure S7. There was a significant difference between sexes (*t* = 3.90, *df* = 7337.70, *p* < .001, *d* = −0.09, 95 % confidence intervals [-0.13, −0.04]), wherein females had an older mean predicted brain age. There was no correlation between BAG and chronological age (*r* = -.02, *p* = .171, 95 % CI [-0.03, 0.01]; Figure S8), indicating no age bias (Treder et al., 2021). BAG was *z*-score normalized prior to analysis.

### 3.3 Mediation models

All adversity dimensions were positively associated with later internalizing and externalizing problems (Table 2). These effects remained at 99 % CI, and most also remained in sensitivity analyses (Table S9). BAG was positively associated with externalizing problems in all models at 95 % and 99 % CIs, and were present in sensitivity analyses. There were no indirect effects of adversity exposure to mental health problems via BAG (Table S10).

**Table 2:** Bayesian Regression Posterior Estimates for Direct Pathways in Mediation Models.

Predictor	Adversity - Brain Age Gap (path *a*)					Brain Age Gap - MH Problems (path *b*)					Adversity - MH Problems (path *c’*)			
*Outcome*	Est	Err	95 % CI	99 % CI		Est	Err	95 % CI	99 % CI		Est	Err	95 % CI	99 % CI
Threat														
*Internalizing*	0.0089	0.0098	-0.0105, 0.0280	-0.0165, 0.0333		0.0143	0.0155	-0.0161, 0.0449	-0.0265, 0.0540		**0.2288**	0.0127	0.2041, 0.2541	0.1969, 0.2628
*Externalizing*	0.0110	0.0098	-0.0081, 0.0301	-0.0146, 0.0361		**0.0564**	0.0175	0.0216, 0.0902	0.0116, 0.1010		**0.2506**	0.0144	0.2225, 0.2788	0.2141, 0.2884
Deprivation														
*Internalizing*	-0.0058	0.0095	-0.0244, 0.0129	-0.0307, 0.0189		0.0239	0.0157	-0.0072, 0.0543	-0.0160, 0.0646		**0.0853**	0.0113	0.0634, 0.1078	0.0565, 0.1154
*Externalizing*	-0.0049	0.0093	-0.0232, 0.0135	-0.0292, 0.0187		**0.0596**	0.0175	0.0258, 0.0940	0.0129, 0.1037		**0.1158**	0.0130	0.0904, 0.1409	0.0813, 0.1490
SES														
*Internalizing*	0.0062	0.0112	-0.0159, 0.0282	-0.0222, 0.0350		0.0240	0.0157	-0.0065, 0.0547	-0.0162, 0.0644		**0.0780**	0.0168	0.0444, 0.1107	0.0346, 0.1207
*Externalizing*	0.0065	0.0112	-0.0154, 0.0283	-0.0226, 0.0363		**0.0597**	0.0172	0.0263, 0.0936	0.0160, 0.1035		**0.1621**	0.0207	0.1218, 0.2022	0.1082, 0.2130
Instability														
*Internalizing*	0.0111	0.0099	-0.0084, 0.0304	-0.0150, 0.0358		0.0166	0.0154	-0.0134, 0.0472	-0.0228, 0.0572		**0.2499**	0.0135	0.2233, 0.2765	0.2147, 0.2846
*Externalizing*	0.0116	0.0099	-0.0078, 0.0310	-0.0140, 0.0366		**0.0514**	0.0173	0.0181, 0.0854	0.0084, 0.0951		**0.3212**	0.0159	0.2898, 0.3527	0.2805, 0.3632

*Note.* Bayesian mixed-effects regression models were used to estimate associations between adversity dimensions (threat, deprivation, socioeconomic stressors [SES], and household instability), brain-age-gap, and mental health problems [MH Problems] (internalizing and externalizing). Each row of the table represents a separate model. Est, mean of the posterior distribution; Err, standard deviation of the posterior distribution; CI, credibility intervals calculated as the lower and upper bounds of 95 % and 99 % quantiles of the posterior distribution.

### 3.4 Sex moderated mediation models

**Fig. 3:**
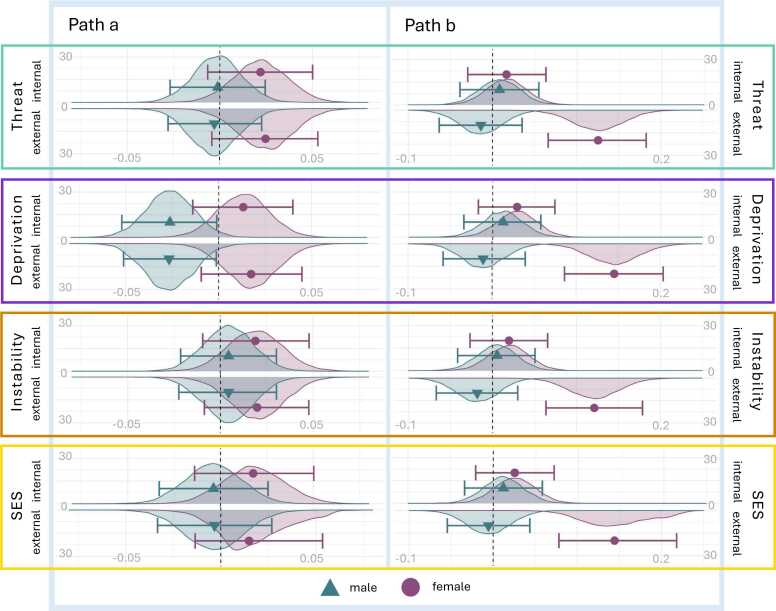
*Bayesian Posterior Distributions from Models Testing Brain-Age-Gap as a Mediator of Dimensional Adversity and Mental Health Problems in Males and Females. Note.* Path a = Adversity as a predictor of brain-age-gap within full mediation model; Path b = Mental health problems (internalizing or externalizing) as a predictor of brain-age-gap within full mediation model. Black dashed vertical line signifies zero. Position of shape along *x* axis signifies the median of the posterior distribution. Error bars signify 95 % credible intervals. Error bars including zero indicate no effect.

Exploratory moderation analyses examined sex differences in model pathways. Specifically, sex was tested as a potential moderator of the relationship between adversity and BAG (first-stage moderation) and separately as a moderator of the relationship between BAG with mental health problems (second-stage moderation) (Figure S5). First-stage moderated mediation was found for models from deprivation and SES to externalizing problems (Table 3). Second-stage moderated mediation was found for the threat to externalizing problems model. Post-hoc mediation analyses conducted within males and females separately failed to identify indirect effects in either sex but did identify differences on specific pathways (Table S12). As illustrated in Fig. 3, the sex moderated mediation reflects higher levels of adversity associated with more negative BAG in males and comparatively more positive BAG in females (first-stage). Further, there was a positive relationship between BAG and externalizing problems in females, which was not present in males (second-stage).

**Table 3:** Sex Moderated Mediation Models.

		First-Stage Moderated Mediation						Second-Stage Moderated Mediation				
		Est	95 %CI L	95 %CI U	99 %CI L	99 %CI U		Est	95 %CI L	95 %CI U	99 %CI L	99 %CI U
Threat												
	*Internalising*	0.00095	-0.00040	0.00314	-0.00096	0.00416		0.00064	-0.00061	0.00241	-0.00114	0.00317
	*Externalising*	0.00323	-0.00040	0.00760	-0.00151	0.00917		**0.00214**	0.00006	0.00512	-0.00053	0.00633
Deprivation												
	*Internalising*	0.00154	-0.00008	0.00405	-0.00055	0.00533		0.00039	-0.00046	0.00180	-0.00089	0.00249
	*Externalising*	**0.00426**	0.00040	0.00905	-0.00079	0.01107		0.00129	-0.00082	0.00398	-0.00151	0.00518
SES												
	*Internalising*	0.00151	-0.00010	0.00418	-0.00062	0.00518		0.00079	-0.00038	0.00271	-0.00080	0.00359
	*Externalising*	**0.00399**	0.00003	0.00869	-0.00119	0.01048		0.00201	-0.00021	0.00511	-0.00103	0.00652
Instability												
	*Internalising*	0.00066	-0.00064	0.00261	-0.00124	0.00355		0.00067	-0.00036	0.00235	-0.00079	0.00311
	*Externalising*	0.00193	-0.00188	0.00610	-0.00323	0.00782		0.00185	-0.00050	0.00491	-0.00136	0.00615

*Note*. First-Stage Moderated Mediation, moderating effect of sex on the association between adversity exposure and brain-age-gap in a model where brain-age-gap mediates adversity and mental health problems; Second-Stage Moderated Mediation, moderating effect of sex on the association between brain-age-gap and mental health problems in a model where brain-age-gap mediates adversity and mental health problems. Est, Estimate; CI L, lower bound credibility interval; CI U, upper bound credibility interval.

## 4 Discussion

This study investigated whether individual differences in the pace of brain development mediated associations between ELA and adolescent mental health problems. While our analysis did not support mediation via brain aging across adversity dimensions, exploratory findings revealed sex-moderation on these pathways. Males exposed to psychosocial deprivation and lower SES exhibited comparatively negative BAG (indicative of neural morphology more consistent with someone of a younger age), whereas females showed more positive BAG (indicative of an older appearing brain). Similarly, threat exposure was associated with more externalizing problems in females with older appearing brain phenotypes, while males displayed the opposite pattern.

Contrary to our hypothesis that older appearing neural phenotypes would predict internalizing symptoms, we found that positive BAG was associated with externalizing problems at three-year follow-up (although the strength of this association was quite low). An association between externalizing problems and BAG aligns with Niu et al. (2022), who reported a positive association between oppositional defiant disorder and the BAG of a cluster of cortical and cerebellar regions. Additionally, Blok et al. (2022) found that externalizing problems were associated with individual deviations (measured as z-scores) in more brain regions than internalizing problems when using an age-normed model of regional brain development. More diffuse regional associations could explain why our whole-brain measure was associated with externalizing but not internalizing problems. Earlier-maturing youth (as measured by pubertal timing) tend to associate with older peers (Negriff et al., 2011). This may increase their exposure to what may be considered normative risk-taking behaviors for older youth but may manifest in externalizing problems in younger but more physically mature adolescents (Vijayakumar and Whittle, 2023).

We also found sex moderation, such that there was a positive association between BAG and externalizing problems in females that was not present in males when examined in separate models. Based on the limited existing literature, it is unclear why such a sex difference might occur. Although sex was accounted for in the brain-age model, it is possible that this result reflects the greater proportion of females with older appearing neural phenotypes in the sample. This finding would benefit from replication with a wider age sample or sex-specific brain-age models.

Evolutionary-developmental theories suggest that sex differences in developmental responses to ELA reflect evolutionary selection pressures related to reproductive fitness (Del Giudice, 2014). Sex-specific selection pressures might explain why males with reduced psychosocial and material resources demonstrated younger appearing brain structure compared to females in our study. Similar sex-specific patterns have been observed in pubertal development, with studies indicating more delayed pubertal timing in males exposed to deprivation (Colich et al., 2020) and threat (Stenson et al., 2021), but accelerated development in females. Notably, in a similar ABCD sample, we previously found accelerated pubertal development following threat for both sexes (Shaul et al., 2024) and Beck et al. (2025) identified a more negative BAG at baseline in males and females exposed to greater psychosocial deprivation (although potential sex differences were not explored). Previous studies have identified sex differences in ELA-associated brain morphology, both for broad adversity (Kelly et al., 2016, White and Kaffman, 2019) and specific types of adversity exposure (Everaerd et al., 2016, Helpman et al., 2017, Teicher et al., 2018). However, interpreting these as accelerations or delays remains challenging given the complexity of normative adolescent neural development. Importantly, the sex-based analyses in this study were exploratory in nature and demonstrated relatively small effect sizes thus they should be interpreted with caution. However, their consistency with previous studies suggests that it is an area that warrants further investigation.

Our analysis revealed no associations between adversity dimensions and BAG across the combined sex sample, contradicting both our hypothesis and some existing literature linking traumatic experiences to older brain age and related biological aging markers (Colich et al., 2020, Drobinin et al., 2022, Gur et al., 2019, Sumner et al., 2019). Contextualization of the current null findings is hindered by the methodological variability across the brain age and adversity literature. For instance, Beck et al. (2025) used ABCD data and 10 adversity dimensions derived from a data-driven factor analysis (Brieant et al., 2023). This study found older appearing brains were associated with greater exposure to an adversity dimension similar to the theory-informed threat dimension of this study. This apparent discrepancy could be due to the different operationalisation of adversity or brain age prediction models (Jirsaraie et al., 2023). Our study employed a model trained on a lifespan sample, whereas many comparable studies used models restricted to adolescence. This may inadequately capture age-related variance in neural development, particularly in regions with non-linear developmental trajectories (de Lange et al., 2022).

### 4.1 Strengths and limitations

In addition to accounting for age variance across the lifespan, a strength of the brain age prediction model used is its public availability, which may facilitate reproducibility for future studies. However, a limitation of this model is that it cannot be used to calculate relative contributions of brain regions and morphometric features to the predictive model. Regional brain development in adolescence follows largely non-linear, asynchronous and sexually dimorphic developmental trajectories (Bethlehem et al., 2022, Forde et al., 2020, Herting et al., 2018, Tamnes et al., 2017, Vijayakumar et al., 2016). A global BAG score aggregates these regions into a single metric, which may mask regional asynchronies in development (Whitmore and Beck, 2025). This is an important consideration for research examining the relationship between ELA and neural development, as different types of adverse exposures may differentially affect specific neural circuitry (Colich et al., 2020, Keding et al., 2021, McLaughlin et al., 2019). For instance, accelerated maturation in some regions could be offset by delayed maturation in others, resulting in a global score that appears normative despite meaningful region-specific variation. Regional brain age models have demonstrated greater sensitivity to individual differences in cognitive and sensorimotor function (Riccardi et al., 2025) and neural correlates of schizophrenia (Zhu et al., 2023) than global metrics in adult populations. Future studies could employ regional brain age models to examine whether adversity dimensions are associated with distinct patterns of regional brain maturation, and whether these differ by sex.

Another strength of this study was the use of data across multiple timepoints, which provides a degree of temporal distinction between the measures of adversity exposure, brain maturation, and mental health outcomes. However, our BAG was calculated on cross-sectional neuroimaging data, which limits our ability to interpret whether observed deviations reflect altered maturational processes (i.e., accelerations or delays in neural development) and the stability of these differences. For example, Rakesh et al. (2021) found that neighbourhood disadvantage was positively associated with BAG in early adolescence, but this was no longer present in late adolescence. Studies with BAG data at multiple timepoints across development would be needed to determine whether deviations represent true differences in maturational tempo and whether such deviations are stable or transient (Parsons and McCormick, 2024). Future studies could utilize forthcoming ABCD data to replicate this study and address this limitation.

The sociodemographic characteristics of our sample (predominately non-Hispanic white and upper-middle socioeconomic status) poses another limitation to the generalizability of our findings beyond similarly stratified populations. Adversity may differentially impact the pace of development for different racial and ethnic populations (Stenson et al., 2021), which remains an important area of future research. Furthermore, while our adversity measures captured experiences occurring prior to baseline assessment (ages 9–11 years), we were unable to determine the precise age, duration, or frequency of exposure. Existing literature indicates that the effect of adversity on neural development exhibits sensitivity to the type and developmental timing of the exposure (Gee and Cohodes, 2023). Additionally, brain age is influenced by genetics (Brouwer et al., 2021), perinatal and postnatal factors such as birth weight (Vidal-Pineiro et al., 2021), while structural adolescent brain development is also driven by puberty-related hormonal shifts (Vijayakumar et al., 2021). Understanding the complex interplay among these multifaceted factors remains an important domain for continued empirical investigation. Finally, for the majority of participants in this study, mental health outcomes were measured during the COVID-19 pandemic. While this was accounted for in the regression models, it may limit the generalizability of these findings beyond the pandemic context.

### 4.2 Conclusion

In summary, structural phenotypic neural maturation – as measured using global BAG – did not explain the relationship between dimensions of ELA and later mental health problems. However, the present findings highlight previously unexamined sex differences in these pathways. Males may be more likely to demonstrate a younger neural phenotype than females when exposed to more psychosocially deprived early-life environments. Meanwhile, females with older appearing neural phenotypes may be most at risk of later externalizing problems. More research is needed to understand how sex differences might manifest in the relationship between ELA and adolescent neural developmental pacing longitudinally.

## CRediT authorship contribution statement

**Nandita Vijayakumar:** Writing – review & editing, Supervision, Resources, Methodology, Formal analysis, Conceptualization. **Michelle Shaul:** Writing – review & editing, Writing – original draft, Visualization, Project administration, Methodology, Formal analysis, Conceptualization. **Sarah Whittle:** Writing – review & editing, Supervision, Resources, Methodology, Conceptualization. **Niousha Dehestani:** Writing – review & editing, Formal analysis, Conceptualization. **Silk Timothy:** Writing – review & editing, Supervision, Conceptualization.

## Declaration of Competing Interest

The authors declare the following financial interests/personal relationships which may be considered as potential competing interests: co-author is an associate editor for this journal - SW. If there are other authors, they declare that they have no known competing financial interests or personal relationships that could have appeared to influence the work reported in this paper.

## Data Availability

The authors do not have permission to share data.

## References

[bib1] Achenbach T.M. (2009).

[bib2] Bath K.G. (2020). Synthesizing Views to Understand Sex Differences in Response to Early Life Adversity. Trends Neurosci..

[bib3] Beck D., Whitmore L., MacSweeney N., Brieant A., Karl V., de Lange A.G., Westlye L.T., Mills K.L., Tamnes C.K. (2025). Dimensions of Early-Life Adversity Are Differentially Associated With Patterns of Delayed and Accelerated Brain Maturation. Biol. Psychiatry.

[bib4] Bellis M.A., Hughes K., Ford K., Ramos Rodriguez G., Sethi D., Passmore J. (2019). Life course health consequences and associated annual costs of adverse childhood experiences across Europe and North America: a systematic review and meta-analysis. Lancet Public Health.

[bib5] Bethlehem, R.A.I., Seidlitz, J., White, S.R., Vogel, J.W., Anderson, K.M., Adamson, C., Adler, S., Alexopoulos, G.S., Anagnostou, E., Areces-Gonzalez, A., Astle, D.E., Auyeung, B., Ayub, M., Ball, G., Baron-Cohen, S., Beare, R., Bedford, S.A., Benegal, V., Beyer, F., … Alexander-Bloch, A.F. (2022). Brain Chart for the Human Lifespan. bioRxiv. 10.1101/2021.06.08.447489.

[bib6] Blok E., Geenjaar E.P.T., Geenjaar E.A.W., Calhoun V.D., White T. (2022). Neurodevelopmental Trajectories in Children With Internalizing, Externalizing and Emotion Dysregulation Symptoms. Front Psychiatry.

[bib7] Brieant A., Vannucci A., Nakua H., Harris J., Lovell J., Brundavanam D., Tottenham N., Gee D.G. (2023). Characterizing the dimensional structure of early-life adversity in the Adolescent Brain Cognitive Development (ABCD) Study. Dev. Cogn. Neurosci..

[bib8] Brouwer R.M., Schutte J., Janssen R., Boomsma D.I., Hulshoff Pol H.E., Schnack H.G. (2021). The Speed of Development of Adolescent Brain Age Depends on Sex and Is Genetically Determined. Cereb. Cortex.

[bib9] Casey B.J., Cannonier T., Conley M.I., Cohen A.O., Barch D.M., Heitzeg M.M., Soules M.E., Teslovich T., Dellarco D.V., Garavan H., Orr C.A., Wager T.D., Banich M.T., Speer N.K., Sutherland M.T., Riedel M.C., Dick A.S., Bjork J.M., Thomas K.M., Workgroup A.I.A. (2018). The Adolescent Brain Cognitive Development (ABCD) study: Imaging acquisition across 21 sites. Dev. Cogn. Neurosci..

[bib10] Chahal R., Miller J.G., Yuan J.P., Buthmann J.L., Gotlib I.H. (2022). An exploration of dimensions of early adversity and the development of functional brain network connectivity during adolescence: Implications for trajectories of internalizing symptoms. Dev. Psychopathol..

[bib11] Chung Y., Addington J., Bearden C.E., Cadenhead K., Cornblatt B., Mathalon D.H., McGlashan T., Perkins D., Seidman L.J., Tsuang M., Walker E., Woods S.W., McEwen S., van Erp T.G.M., Cannon T.D., North American Prodrome Longitudinal Study, C., the Pediatric Imaging, N., & Genetics Study, C (2018). Use of Machine Learning to Determine Deviance in Neuroanatomical Maturity Associated With Future Psychosis in Youths at Clinically High Risk. JAMA Psychiatry.

[bib12] Cohen J.W., Ramphal B., DeSerisy M., Zhao Y., Pagliaccio D., Colcombe S., Milham M.P., Margolis A.E. (2024). Relative brain age is associated with socioeconomic status and anxiety/depression problems in youth. Dev. Psychol..

[bib13] Cole J.H., Franke K. (2017). Predicting Age Using Neuroimaging: Innovative Brain Ageing Biomarkers. Trends Neurosci..

[bib14] Colich N.L., Rosen M.L., Williams E.S., McLaughlin K.A. (2020). Biological aging in childhood and adolescence following experiences of threat and deprivation: A systematic review and meta-analysis. Psychol. Bull..

[bib15] Cropley V.L., Tian Y., Fernando K., Mansour L.S., Pantelis C., Cocchi L., Zalesky A. (2021). Brain-Predicted Age Associates With Psychopathology Dimensions in Youths. Biol. Psychiatry Cogn. Neurosci. Neuroimaging.

[bib16] Dehestani N., Whittle S., Vijayakumar N., Silk T.J. (2023). Developmental brain changes during puberty and associations with mental health problems. Dev. Cogn. Neurosci..

[bib17] Del Giudice M. (2014). An Evolutionary Life History Framework for Psychopathology. Psychol. Inq..

[bib18] Donovan A., Assari S., Grella C., Shaheen M., Richter L., Friedman T.C. (2024). Neuroendocrine mechanisms in the links between early life stress, affect, and youth substance use: A conceptual model for the study of sex and gender differences. Front Neuroendocr..

[bib19] Drobinin V., Van Gestel H., Helmick C.A., Schmidt M.H., Bowen C.V., Uher R. (2022). The Developmental Brain Age Is Associated With Adversity, Depression, and Functional Outcomes Among Adolescents. Biol. Psychiatry Cogn. Neurosci. Neuroimaging.

[bib20] Ellis B.J., Figueredo A.J., Brumbach B.H., Schlomer G.L. (2009). Fundamental Dimensions of Environmental Risk: The Impact of Harsh versus Unpredictable Environments on the Evolution and Development of Life History Strategies. Hum. Nat..

[bib21] Ellis B.J., Sheridan M.A., Belsky J., McLaughlin K.A. (2022). Why and how does early adversity influence development? Toward an integrated model of dimensions of environmental experience. Dev. Psychopathol..

[bib22] Everaerd D., Klumpers F., Zwiers M., Guadalupe T., Franke B., van Oostrom I., Schene A., Fernandez G., Tendolkar I. (2016). Childhood abuse and deprivation are associated with distinct sex-dependent differences in brain morphology. Neuropsychopharmacology.

[bib23] Forde N.J., Jeyachandra J., Joseph M., Jacobs G.R., Dickie E., Satterthwaite T.D., Shinohara R.T., Ameis S.H., Voineskos A.N. (2020). Sex Differences in Variability of Brain Structure Across the Lifespan. Cereb. Cortex.

[bib24] Franke K., Gaser C. (2019). Ten Years of BrainAGE as a Neuroimaging Biomarker of Brain Aging: What Insights Have We Gained?. Front Neurol..

[bib25] Garavan H., Bartsch H., Conway K., Decastro A., Goldstein R.Z., Heeringa S., Jernigan T., Potter A., Thompson W., Zahs D. (2018). Recruiting the ABCD sample: Design considerations and procedures. Dev. Cogn. Neurosci..

[bib26] Gee D.G. (2021). Early Adversity and Development: Parsing Heterogeneity and Identifying Pathways of Risk and Resilience. Am. J. Psychiatry.

[bib27] Gee D.G., Cohodes E.M. (2023). Leveraging the developmental neuroscience of caregiving to promote resilience among youth exposed to adversity. Dev. Psychopathol..

[bib28] Gelman A., Simpson D., Betancourt M. (2017). The Prior Can Often Only Be Understood in the Context of the Likelihood. Entropy.

[bib29] Gur R.E., Moore T.M., Rosen A.F.G., Barzilay R., Roalf D.R., Calkins M.E., Ruparel K., Scott J.C., Almasy L., Satterthwaite T.D., Shinohara R.T., Gur R.C. (2019). Burden of Environmental Adversity Associated With Psychopathology, Maturation, and Brain Behavior Parameters in Youths. JAMA Psychiatry.

[bib30] Hagler D.J., Hatton S., Cornejo M.D., Makowski C., Fair D.A., Dick A.S., Sutherland M.T., Casey B.J., Barch D.M., Harms M.P., Watts R., Bjork J.M., Garavan H.P., Hilmer L., Pung C.J., Sicat C.S., Kuperman J., Bartsch H., Xue F., Dale A.M. (2019). Image processing and analysis methods for the Adolescent Brain Cognitive Development Study. NeuroImage.

[bib31] Hayes A.F., Rockwood N.J. (2019). Conditional Process Analysis: Concepts, Computation, and Advances in the Modeling of the Contingencies of Mechanisms. Am. Behav. Sci..

[bib32] Helpman L., Zhu X., Suarez-Jimenez B., Lazarov A., Monk C., Neria Y. (2017). Sex Differences in Trauma-Related Psychopathology: a Critical Review of Neuroimaging Literature (2014-2017). Curr. Psychiatry Rep..

[bib33] Herting M.M., Johnson C., Mills K.L., Vijayakumar N., Dennison M., Liu C., Goddings A.L., Dahl R.E., Sowell E.R., Whittle S., Allen N.B., Tamnes C.K. (2018). Development of subcortical volumes across adolescence in males and females: A multisample study of longitudinal changes. NeuroImage.

[bib34] Hodes G.E., Epperson C.N. (2019). Sex Differences in Vulnerability and Resilience to Stress Across the Life Span. Biol. Psychiatry.

[bib35] Holm M.C., Leonardsen E.H., Beck D., Dahl A., Kjelkenes R., de Lange A.G., Westlye L.T. (2023). Linking brain maturation and puberty during early adolescence using longitudinal brain age prediction in the ABCD cohort. Dev. Cogn. Neurosci..

[bib36] Hu L.-t, Bentler P.M. (1998). Fit indices in covariance structure modeling: Sensitivity to underparameterized model misspecification. Psychol. Methods.

[bib37] Jirsaraie R.J., Gorelik A.J., Gatavins M.M., Engemann D.A., Bogdan R., Barch D.M., Sotiras A. (2023). A systematic review of multimodal brain age studies: Uncovering a divergence between model accuracy and utility. Patterns (N. Y).

[bib38] Kasparek S.W., Gaston-Panthaki A., Hanford L.C., Lengua L.J., Sheridan M.A., McLaughlin K.A. (2023). Does reward processing moderate or mediate the link between childhood adversity and psychopathology: A longitudinal study. Dev. Psychopathol..

[bib39] Keding T.J., Heyn S.A., Russell J.D., Zhu X., Cisler J., McLaughlin K.A., Herringa R.J. (2021). Differential Patterns of Delayed Emotion Circuit Maturation in Abused Girls With and Without Internalizing Psychopathology. Am. J. Psychiatry.

[bib40] Kelly P.A., Viding E., Puetz V.B., Palmer A.L., Samuel S., McCrory E.J. (2016). The sexually dimorphic impact of maltreatment on cortical thickness, surface area and gyrification. J. Neural Transm. (Vienna).

[bib41] Konrad K., Firk C., Uhlhaas P.J. (2013). Brain development during adolescence: neuroscientific insights into this developmental period. Dtsch Arztebl Int.

[bib42] de Lange A.G., Anaturk M., Rokicki J., Han L.K.M., Franke K., Alnaes D., Ebmeier K.P., Draganski B., Kaufmann T., Westlye L.T., Hahn T., Cole J.H. (2022). Mind the gap: Performance metric evaluation in brain-age prediction. Hum. Brain Mapp..

[bib43] Leonardsen E.H., Peng H., Kaufmann T., Agartz I., Andreassen O.A., Celius E.G., Espeseth T., Harbo H.F., Hogestol E.A., Lange A.M., Marquand A.F., Vidal-Pineiro D., Roe J.M., Selbaek G., Sorensen O., Smith S.M., Westlye L.T., Wolfers T., Wang Y. (2022). Deep neural networks learn general and clinically relevant representations of the ageing brain. NeuroImage.

[bib44] Lopez C.M., Andrews A.R., Chisolm A.M., de Arellano M.A., Saunders B., Kilpatrick D.G. (2017). Racial/ethnic differences in trauma exposure and mental health disorders in adolescents. Cult. Divers. Ethn. Minor. Psychol..

[bib45] Luna A., Bernanke J., Kim K., Aw N., Dworkin J.D., Cha J., Posner J. (2021). Maturity of gray matter structures and white matter connectomes, and their relationship with psychiatric symptoms in youth. Hum. Brain Mapp..

[bib46] Lund M.J., Alnaes D., de Lange A.G., Andreassen O.A., Westlye L.T., Kaufmann T. (2022). Brain age prediction using fMRI network coupling in youths and associations with psychiatric symptoms. Neuroimage Clin..

[bib47] Lyons C.E., Razzoli M., Bartolomucci A. (2023). The impact of life stress on hallmarks of aging and accelerated senescence: Connections in sickness and in health. Neurosci. Biobehav. Rev..

[bib48] MacSweeney N., Beck D., Whitmore L., Mills K.L., Westlye L.T., von Soest T., Ferschmann L., Tamnes C.K. (2025). Multimodal Brain Age Indicators of Internalizing Problems in Early Adolescence: A Longitudinal Investigation. Biol. Psychiatry Cogn. Neurosci. Neuroimaging.

[bib49] McLaughlin K.A., Sheridan M.A. (2016). Beyond Cumulative Risk: A Dimensional Approach to Childhood Adversity. Curr. Dir. Psychol. Sci..

[bib50] McLaughlin K.A., Sheridan M.A., Lambert H.K. (2014). Childhood adversity and neural development: deprivation and threat as distinct dimensions of early experience. Neurosci. Biobehav. Rev..

[bib51] McLaughlin K.A., Weissman D., Bitran D. (2019). Childhood Adversity and Neural Development: A Systematic Review. Annu. Rev. Dev. Psychol..

[bib52] McLaughlin K.A., Colich N.L., Rodman A.M., Weissman D.G. (2020). Mechanisms linking childhood trauma exposure and psychopathology: a transdiagnostic model of risk and resilience. BMC Med..

[bib53] McLaughlin K.A., Sheridan M.A., Humphreys K.L., Belsky J., Ellis B.J. (2021). The Value of Dimensional Models of Early Experience: Thinking Clearly About Concepts and Categories. Perspect. Psychol. Sci..

[bib54] Miller A.B., Sheridan M.A., Hanson J.L., McLaughlin K.A., Bates J.E., Lansford J.E., Pettit G.S., Dodge K.A. (2018). Dimensions of deprivation and threat, psychopathology, and potential mediators: A multi-year longitudinal analysis. J. Abnorm. Psychol..

[bib55] Murgueitio N., Sheridan M.A., Bauer D.J., Propper C.B. (2024). Developmental mechanisms linking deprivation and threat to psychopathology and school outcomes. Dev. Psychopathol..

[bib56] Negriff S., Susman E.J., Trickett P.K. (2011). The developmental pathway from pubertal timing to delinquency and sexual activity from early to late adolescence. J. Youth Adolesc..

[bib57] Niu X., Taylor A., Shinohara R.T., Kounios J., Zhang F. (2022). Multidimensional brain-age prediction reveals altered brain developmental trajectory in psychiatric disorders. Cereb. Cortex.

[bib58] Parsons S., McCormick E.M. (2024). Limitations of two time point data for understanding individual differences in longitudinal modeling - What can difference reveal about change?. Dev. Cogn. Neurosci..

[bib59] Rakesh D., Cropley V., Zalesky A., Vijayakumar N., Allen N.B., Whittle S. (2021). Neighborhood disadvantage and longitudinal brain-predicted-age trajectory during adolescence. Dev. Cogn. Neurosci..

[bib60] Riccardi N., Teghipco A., Newman-Norlund S., Newman-Norlund R., Rangus I., Rorden C., Fridriksson J., Bonilha L. (2025). Distinct brain age gradients across the adult lifespan reflect diverse neurobiological hierarchies. Commun. Biol..

[bib61] RStudio Team (2020). In RStudio.

[bib62] Schafer J.L., McLaughlin K.A., Manfro G.G., Pan P., Rohde L.A., Miguel E.C., Simioni A., Hoffmann M.S., Salum G.A. (2022). Threat and deprivation are associated with distinct aspects of cognition, emotional processing, and psychopathology in children and adolescents. Dev. Sci..

[bib63] Shaul M., Whittle S., Silk T.J., Vijayakumar N. (2024). Pubertal timing mediates the association between threat adversity and psychopathology. Psychol. Med..

[bib64] Shonkoff J.P., Slopen N., Williams D.R. (2021). Early Childhood Adversity, Toxic Stress, and the Impacts of Racism on the Foundations of Health. Annu. Rev. Public Health.

[bib65] Stenson A.F., Michopoulos V., Stevens J.S., Powers A., Jovanovic T. (2021). Sex-Specific Associations Between Trauma Exposure, Pubertal Timing, and Anxiety in Black Children. Front. Hum. Neurosci..

[bib66] Sumner J.A., Colich N.L., Uddin M., Armstrong D., McLaughlin K.A. (2019). Early Experiences of Threat, but Not Deprivation, Are Associated With Accelerated Biological Aging in Children and Adolescents. Biol. Psychiatry.

[bib67] Tamnes C.K., Herting M.M., Goddings A.L., Meuwese R., Blakemore S.J., Dahl R.E., Guroglu B., Raznahan A., Sowell E.R., Crone E.A., Mills K.L. (2017). Development of the Cerebral Cortex across Adolescence: A Multisample Study of Inter-Related Longitudinal Changes in Cortical Volume, Surface Area, and Thickness. J. Neurosci..

[bib68] Teicher M.H., Anderson C.M., Ohashi K., Khan A., McGreenery C.E., Bolger E.A., Rohan M.L., Vitaliano G.D. (2018). Differential effects of childhood neglect and abuse during sensitive exposure periods on male and female hippocampus. NeuroImage.

[bib69] Treder M.S., Shock J.P., Stein D.J., du Plessis S., Seedat S., Tsvetanov K.A. (2021). Correlation Constraints for Regression Models: Controlling Bias in Brain Age Prediction. Front Psychiatry.

[bib70] Truelove-Hill M., Erus G., Bashyam V., Varol E., Sako C., Gur R.C., Gur R.E., Koutsouleris N., Zhuo C., Fan Y., Wolf D.H., Satterthwaite T.D., Davatzikos C. (2020). A Multidimensional Neural Maturation Index Reveals Reproducible Developmental Patterns in Children and Adolescents. J. Neurosci..

[bib71] Usacheva M., Choe D., Liu S., Timmer S., Belsky J. (2022). Testing the empirical integration of threat-deprivation and harshness-unpredictability dimensional models of adversity. Dev. Psychopathol..

[bib72] Van Buuren S. (2012). Flexible Imputation of Missing Data.

[bib73] Vehtari, A. (2024, *Prior Choice Recommendations*. Retrieved 7 May 2024 from 〈https://github.com/stan-dev/stan/wiki/Prior-Choice-Recommendations〉.

[bib74] Vidal-Pineiro D., Wang Y., Krogsrud S.K., Amlien I.K., Baare W.F.C., Bartres-Faz D., Bertram L., Brandmaier A.M., Drevon C.A., Duzel S., Ebmeier K., Henson R.N., Junque C., Kievit R.A., Kuhn S., Leonardsen E., Lindenberger U., Madsen K.S., Magnussen F., Fjell A. (2021). Individual variations in 'brain age' relate to early-life factors more than to longitudinal brain change. Elife.

[bib75] Vijayakumar N., Whittle S. (2023). A systematic review into the role of pubertal timing and the social environment in adolescent mental health problems. Clin. Psychol. Rev..

[bib76] Vijayakumar N., Allen N.B., Youssef G., Dennison M., Yucel M., Simmons J.G., Whittle S. (2016). Brain development during adolescence: A mixed-longitudinal investigation of cortical thickness, surface area, and volume. Hum. Brain Mapp..

[bib77] Vijayakumar N., Youssef G.J., Allen N.B., Anderson V., Efron D., Hazell P., Mundy L., Nicholson J.M., Patton G., Seal M.L., Simmons J.G., Whittle S., Silk T. (2021). A longitudinal analysis of puberty-related cortical development. NeuroImage.

[bib78] Vogel S.C., Perry R.E., Brandes-Aitken A., Braren S., Blair C. (2021). Deprivation and threat as developmental mediators in the relation between early life socioeconomic status and executive functioning outcomes in early childhood. Dev. Cogn. Neurosci..

[bib79] White J.D., Kaffman A. (2019). The Moderating Effects of Sex on Consequences of Childhood Maltreatment: From Clinical Studies to Animal Models. Front Neurosci..

[bib80] Whitmore L., Beck D. (2025). Current challenges and future directions for brain age prediction in children and adolescents. Nat. Commun..

[bib81] Young E.S., Frankenhuis W.E., Ellis B.J. (2020). Theory and measurement of environmental unpredictability. Evol. Hum. Behav..

[bib82] Yuan Y., MacKinnon D.P. (2009). Bayesian mediation analysis. Psychol. Methods.

[bib83] Zhou Z., Li Y., Zhang Y., Liu J., Ai H., Liu M., Qiu J., Luo Y.J., Xu P. (2023). Differential effects of generalized anxiety and separation anxiety on brain structural development during adolescence. J. Affect Disord..

[bib84] Zhu J.D., Wu Y.F., Tsai S.J., Lin C.P., Yang A.C. (2023). Investigating brain aging trajectory deviations in different brain regions of individuals with schizophrenia using multimodal magnetic resonance imaging and brain-age prediction: a multicenter study. Transl. Psychiatry.

