# Supplementary material for: Dimensional adversity, brain-age, & mental health: Differences in male and female adolescents
Source: Dev Cogn Neurosci. 2026 Jan 13;78:101671. doi: 10.1016/j.dcn.2026.101671 (PMC12856638; doi:10.1016/j.dcn.2026.101671)
Supplement: Figure S1 — Supplementary material [file mmc1.pdf]

Supplementary Materials

Figure S1

Flow Chart of Participant Exclusion and Analyses

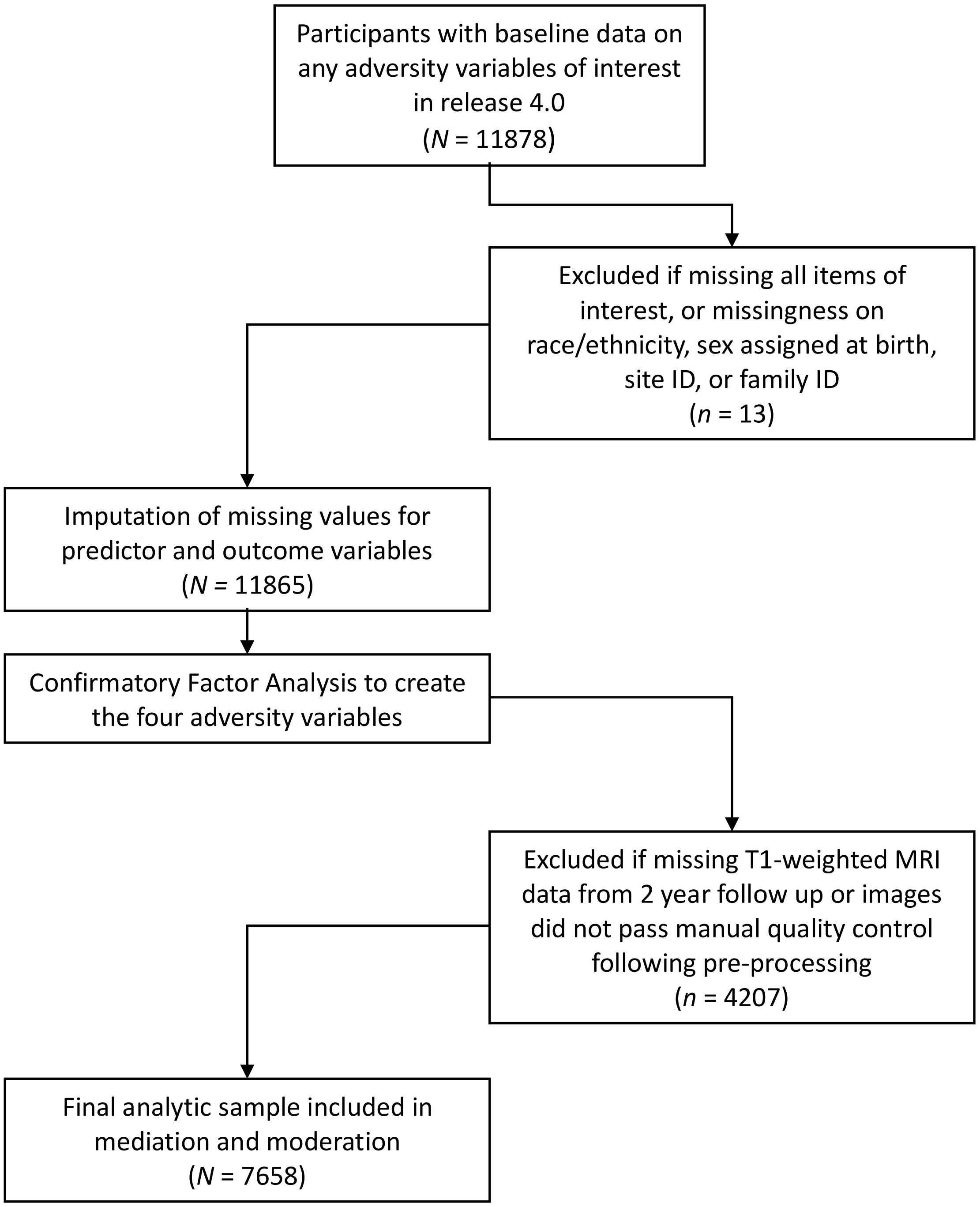

Table S1

Questionnaires from the Adolescent Behaviour and Cognitive Development Study used to Measure Dimensional Adversity Exposure

Questionnaire	Table Name	Details	Informant	Measure
Kiddie Schedule for Affective Disorders and Schizophrenia for DSM-5 (PTSD-Checklist)	abcd_ptsd01	Lifetime occurrence of traumatic events	Parent	0 – "No", 1 – "Yes"
Kiddie Schedule for Affective Disorders and Schizophrenia for DSM-5 (Background)	dibf01	School, family and social relations at baseline	Parent	0 – "No", 1 – "Yes"
Family Environment Scale – Conflict Subscale	abcd_fes01	Perceived degree of conflict expressed by most members of the family at baseline. Some items are reverse coded and have been adjusted so that higher scores reflect greater conflict.	Youth	"True", "False"
Children's Reports of Parental Behavior Inventory	crpbi01	Perceived acceptance from primary and secondary caregivers at baseline	Youth	1 – "Not like them", 3 – "A lot like them"
Parental Monitoring Questionnaire	pmq01	Perceived degree of parental supervision at baseline	Youth	1 – "Never', 5 – "Always"
Demographics Survey	pdem02	Experiences of material hardship within the past 12 months; income bracket of caregivers; education level of caregivers	Parent	0 – "No", 1 – "Yes"; 1 – "Less than $5000", 10 – "$200,000 or greater"; 0 – "Never attended", 21 – "Doctoral degree"
Family History Assessment	abcd_fhxssp01	Summary scores from family history inventory assessing lifetime occurrence of psychological problems in the child's biological relatives	Parent	0 – "No problem endorsed", 1 – "Problem endorsed"
Residential History Derived Scores	abcd_rhds01	External environmental and neighborhood data linked to residential geocodes	*Automated*	*Item specific*
Neighborhood Safety and Crime	abcd_pnsc01	Perceived level of neighborhood safety and crime at baseline	Parent	1 – "Strongly disagree", 5 – "Strongly agree"

Note. All questionnaires taken from Release 4.0. Parent self-report of the attained education level of themselves and a secondary caregiver were measured as an ordinal variable. Lower values represent lower attained education.

Table S2

Allocation of Questionnaire Items to Four Adversity Factors in Confirmatory Factor Analysis

Factor	Adversity Domain	Item	ABCD Item Name (Survey short name)
Threat	Physical Abuse	Beaten to the point of having bruises by a family member	ksads_ptsd_raw_763_p (abcd_ptsd01)
		A non-family member threatened to kill your child	ksads_ptsd_raw_764_p (abcd_ptsd01)
		A family member threatened to kill your child	ksads_ptsd_raw_765_p (abcd_ptsd01)
	Sexual Abuse	A grown up in the home touched your child in their privates, had your child touch their privates, or did other sexual things to your child	ksads_ptsd_raw_767_p (abcd_ptsd01)
		An adult outside your family touched your child in their privates, had your child touch their privates or did other sexual things to your child	ksads_ptsd_raw_768_p (abcd_ptsd01)
		A peer forced your child to do something sexually	ksads_ptsd_raw_769_p (abcd_ptsd01)
	Witnessing Violence	Witness the grownups in the home push, shove or hit one another	ksads_ptsd_raw_766_p (abcd_ptsd01)
		Witnessed or present during an act of terrorism	ksads_ptsd_raw_758_p (abcd_ptsd01)
		Witnessed death or mass destruction in a war zone	ksads_ptsd_raw_759_p (abcd_ptsd01)
		Witnessed someone shot or stabbed in the community	ksads_ptsd_raw_760_p (abcd_ptsd01)
		Family members sometimes get so angry they throw things	fes_youth_q3 (abcd_fes01)
		Family members sometimes hit each other	fes_youth_q6 (abcd_fes01)
	Bullying	Does your child have any problems with bullying at school or in your neighborhood?	kbi_p_c_bully (dibf01)
	Accidental Injury or Death	A car accident in which your child or another person in the car was hurt bad enough to require medical attention?	ksads_ptsd_raw_754_p (abcd_ptsd01)
		Another significant accident for which your child needed specialized and intensive medical treatment?	ksads_ptsd_raw_755_p (abcd_ptsd01)
		Witnessed or caught in a fire that caused significant property damage or personal injury	ksads_ptsd_raw_756_p (abcd_ptsd01)
		Witnessed or caught in a natural disaster that caused significant property damage or personal injury	ksads_ptsd_raw_757_p (abcd_ptsd01)
		Learned about the sudden unexpected death of a loved one	ksads_ptsd_raw_770_p (abcd_ptsd01)
Deprivation	Emotional Neglect	Makes me feel better after talking over my worries with him/her	crpbi_parent1_y; crpbi_caregiver12_y (crpbi01)
		Smiles at me very often	crpbi_parent2_y; crpbi_caregiver13_y (crpbi01)

		Is able to make me feel better when I am upset	crpbi_parent3_y; crpbi_caregiver14_y (crpbi01)
		Believes in showing his/her love for me	crpbi_parent4_y; crpbi_caregiver15_y (crpbi01)
		Is easy to talk to	crpbi_parent5_y; crpbi_caregiver16_y (crpbi01)
		We fight a lot in our family [r]	fes_youth_q1 (abcd_fes01)
		Family members rarely become openly angry	fes_youth_q2 (abcd_fes01)
		Family members hardly ever lose their tempers	fes_youth_q4 (abcd_fes01)
		Family members often criticize each other	fes_youth_q5 (abcd_fes01)
		If there is a disagreement in our family, we try hard to smooth things over and keep the peace [r]	fes_youth_q7 (abcd_fes01)
		Family members often try to one-up or outdo each other [r]	fes_youth_q8 (abcd_fes01)
		In our family, we believe you don't ever get anywhere by raising your voice	fes_youth_q9 (abcd_fes01)
	Physical Neglect	How often do your parents/guardians know where you are?	parent_monitor_q1_y (pmq01)
		How often do your parents know who you are with when you are not at school and away from home?	parent_monitor_q2_y (pmq01)
		If you are at home when your parents or guardians are not, how often do you know how to get in touch with them?	parent_monitor_q3_y (pmq01)
		How often do you talk to your parent or guardian about your plans for the coming day, such as your plans about what will happen at school or what you are going to do with friends?	parent_monitor_q4_y (pmq01)
		In an average week, how many times do you and your parents/guardians, eat dinner together?	parent_monitor_q5_y (pmq01)
Household Instability	Parental Separation	Child lives with non-biological parents or other caregivers at least part of the time, is adopted or in custodial care	kbi_p_c_guard__[3-12] (dibf01); demo_prim (pdem02)
	Caregiver Substance Abuse	Overall parents alcohol problem	famhx_ss_parent_alc_p (abcd_fhxssp01)
		Overall parents drug use problem	famhx_ss_parent_dg_p (abcd_fhxssp01)
	Caregiver Mental Illness	Overall parents depression problem	famhx_ss_parent_dprs_p (abcd_fhxssp01)
		Overall parents mania problem	famhx_ss_parent_ma_p (abcd_fhxssp01)
		Overall parents nerves/nervous breakdown problem	famhx_ss_parent_nrv_p (abcd_fhxssp01)
		Overall parents hospitalized due to emotional/mental problem	famhx_ss_parent_hspd_p (abcd_fhxssp01)

		Overall parents attempted or committed suicide	famhx_ss_parent_scd_p (abcd_fhxssp01)
		Overall parents trouble holds job/fights/police problem	famhx_ss_parent_trb_p (abcd_fhxssp01)
Socio-economic Stressors	Income-to-needs ratio (INR)	Total family income divided by poverty threshold based on household size	demo_comb_income_v2 ; demo_roster_v2 (pdem02)
	Parent education	Mean score of parent and partner highest grade or level of school completed or the highest degree received	demo_prnt_ed_v2_l demo_prtnr_ed_v2_l (pdem02)
	Neighbourhood disadvantage	Area Deprivation Index: national percentiles	reshist_addr1_adi_perc (abcd_rhds01)

Analysis of Missing Data

Table S3

Breakdown of Missing Data for Variables of Interest

	Any Missing		All Missing		Complete		Total Missing
	n	*%*	*n*	*%*	*n*	*%*	*%*
Threat	325	4.24	0	0.00	7333	95.76	3.58
Deprivation	40	0.52	9	0.12	7618	99.48	0.20
SES	1093	14.27	1	0.01	6565	85.73	5.01
Instability	1121	14.64	0	0.00	6537	85.36	4.84
Internalizing	490	6.40	490	6.40	7168	93.60	6.40
Externalizing	490	6.40	490	6.40	7168	93.60	6.40
All	2486	32.50	0	0.00	5172	67.50	2.54

Note. Individual items that formed the adversity dimensions (threat, deprivation, SES, instability) are outlined in Table S1. Internalizing and Externalizing refer to the corresponding Child Behavior Checklist problem scale items (cbcl_scr_syn_internal_r_3yr, cbcl_scr_syn_external_r_3yr). Missing calculated in final analytic sample (*n* = 7658) prior to imputation. Any Missing, participants with data missing from any of the items; All Missing, participants with data missing from all items; Complete, participants with no data missing from items. Total Missing, percentage of missingness for all items across all participants.

Statistical Analyses

Unless otherwise stated, analyses were conducted in *RStudio* (RStudio Team, 2020 [version 2023.06.1+524]) using *R* (R Core Team, 2021 [version 4.3.1]). Code available at https://github.com/MichelleShaul/adversity_brainage.

Imputation

Imputation of missing data was conducted using the *mice* [3.16.0] package (Van Buuren & Groothuis-Oudshoorn, 2011). Baseline adversity items (Table S2), CBCL internalizing and externalizing problems from available timepoints, interview age at available timepoints, sex assigned at birth, race/ethnicity, and COVID-19 pandemic three-year follow-up interview timing were used as predictors. Potential predictor items were assessed using the *flux* function, which quantifies the usefulness of a variable as a predictor for imputing missing data in other variables by providing an 'outflux' value. The outflux value is the proportion of pairings wherein a variable is observed and its pair is missing, divided by the number of incomplete data cells. Based on recommendations by Van Buuren (2012), variables with an outflux <.5 were not used as predictors. Variables not used as predictors included: "crpbi_caregiver12_y", "crpbi_caregiver15_y", "crpbi_caregiver16_y", "crpbi_caregiver14_y", "crpbi_caregiver13_y", "cbcl_scr_syn_internal_r_4yr", "cbcl_scr_syn_external_r_4yr". Predictive mean matching was used to calculate missing values with 10 maximum iterations and 8 imputations, based on the recommendation that the number of imputations should match the percentage of missingness (8.25% incomplete cases; White et al., 2010).

Examination of internalizing and externalizing outcome variables found no meaningful difference between observed and imputed values (Table S4).

Table S4

Comparison of Observed and Imputed Values for Mental Health Problem Items at 3-Year Follow-Up

3-Year Outcome	Observed		Imputed		Comparison
	M(SD)	*Min-Max*	*M(SD)*	*Min-Max*	
CBCL Internalizing	5.10 (5.90)	0-49	5.20 (5.80)	0-49	$F(1,21189) = 1.93$, $p = .165$, $\eta^2 <.001$ [0.00,1.00]
CBCL Externalizing	2.00 (2.40)	0-12	2.00 (2.30)	0-12	$F(1,21189) = 2.50$, $p = .116$, $\eta^2 <.001$ [0.00,1.00]

Brain-Age

The minimally processed T1-weighted MRI images at two-year follow up from the ABCD 5.0 data release were used to calculated predicted brain age (data structure: *fmriresults01*), which was filtered for those recommended for use (*imgincl_t1w_include* variable in the *abcd_imgincl01* data structure). These images had undergone initial quality control and compliance checks as well as intensity scaling and inhomogeneity correction (for details see Hagler et al., 2019). Additional processing was undertaken consistent with methods used by Leonardsen et al. (2022), which included:

1. Transformation to Talairach space, intensity normalization, and skull stripping using the *recon-all* function in *Freesurfer* version 5.3.
2. Conversion of resulting brainmask.mgz files to NIfTI format using *mri-convert*
3. Standard label orientation using *fslorient2std* in *FSL* version 6.0
4. Registration to Montreal Neurological Institute's 152 brain template (MNI152) with 6 degrees of freedom using *FLIRT*
5. Image boarders cropped to 6:173, 2:214 and 0:160 voxels in the sagittal, coronal, and axial directions respectively

A manual quality control check on all images was performed following step 1 to ensure no gross abnormalities (e.g., large areas of brain missing) following the skull stripping, resulting in exclusion of a further 32 participants.

Processed images were then submitted to the SFCN-reg model using *Python* version 3.9.0 code available on GitHub (https://github.com/estenhl/pyment-public). All image processing and model prediction was performed on the *Spartan* High Performance Computing system operated by Research Computing Services at The University of Melbourne.

Bayesian Regression Models

Bayesian regression models were used to test for mediation and moderation. Analysis was conducted using the *brms* package with the syntax for multivariate multilevel models (Bürkner, 2017). To allow the observed data to have greater influence on the estimates, weakly informative conjugate priors were used for all parameters (Gelman et al., 2017; Yuan & MacKinnon, 2009).

Weakly informative Student's *t* priors (μ = 0, σ = 1, ν = 3) were specified for the population-level (fixed effect) parameters and *brms* default priors were deemed appropriate for remaining parameters (Vehtari, 2024). Posterior distributions were estimated with Markov chain Monte Carlo simulations. Samples were derived by 4 Markov chains with 15000 iterations (2000 warm-up samples) and target average acceptance probability of .90. Adequate model convergence was assessed by Gelman-Rubin statistic (Rhat) values < 1.01, Effective Sample Sizes (ESS) values > 400 and visual inspection of the posterior predictive checks (Vehtari et al., 2021), which are presented in Figures S3 and S4.

Figure S2

Histograms of Response Variables in Bayesian Regression Models

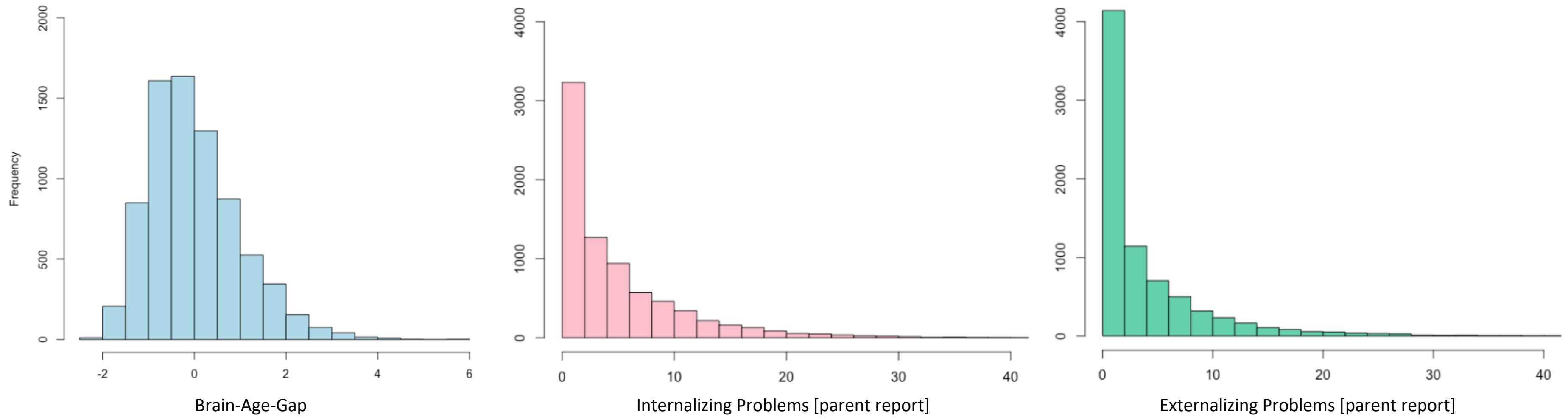

Figure S3

Posterior Predictive Checks for Mediation Models – Density Distributions

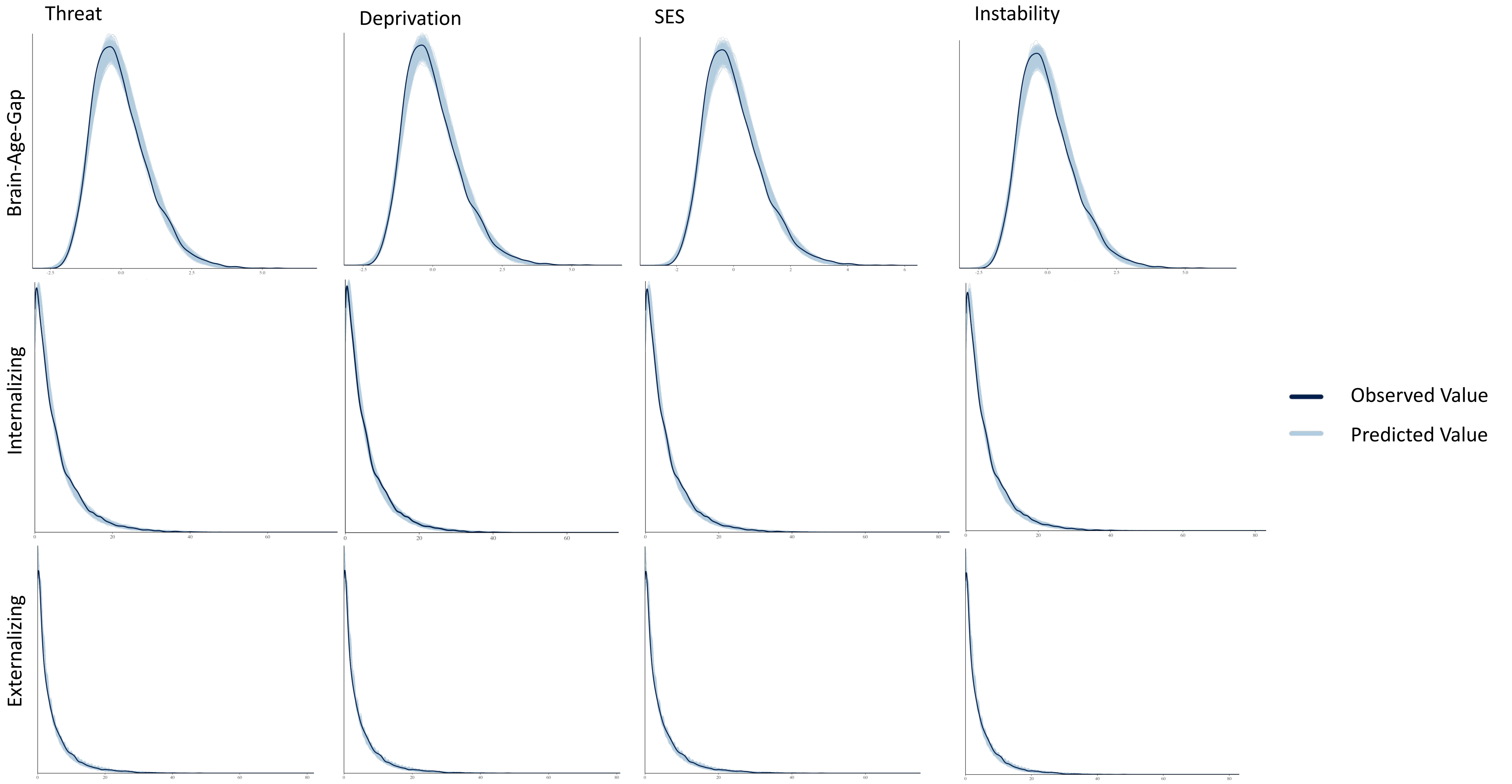

Figure S4

Posterior Predictive Checks for Mediation Models – Predicted Mean

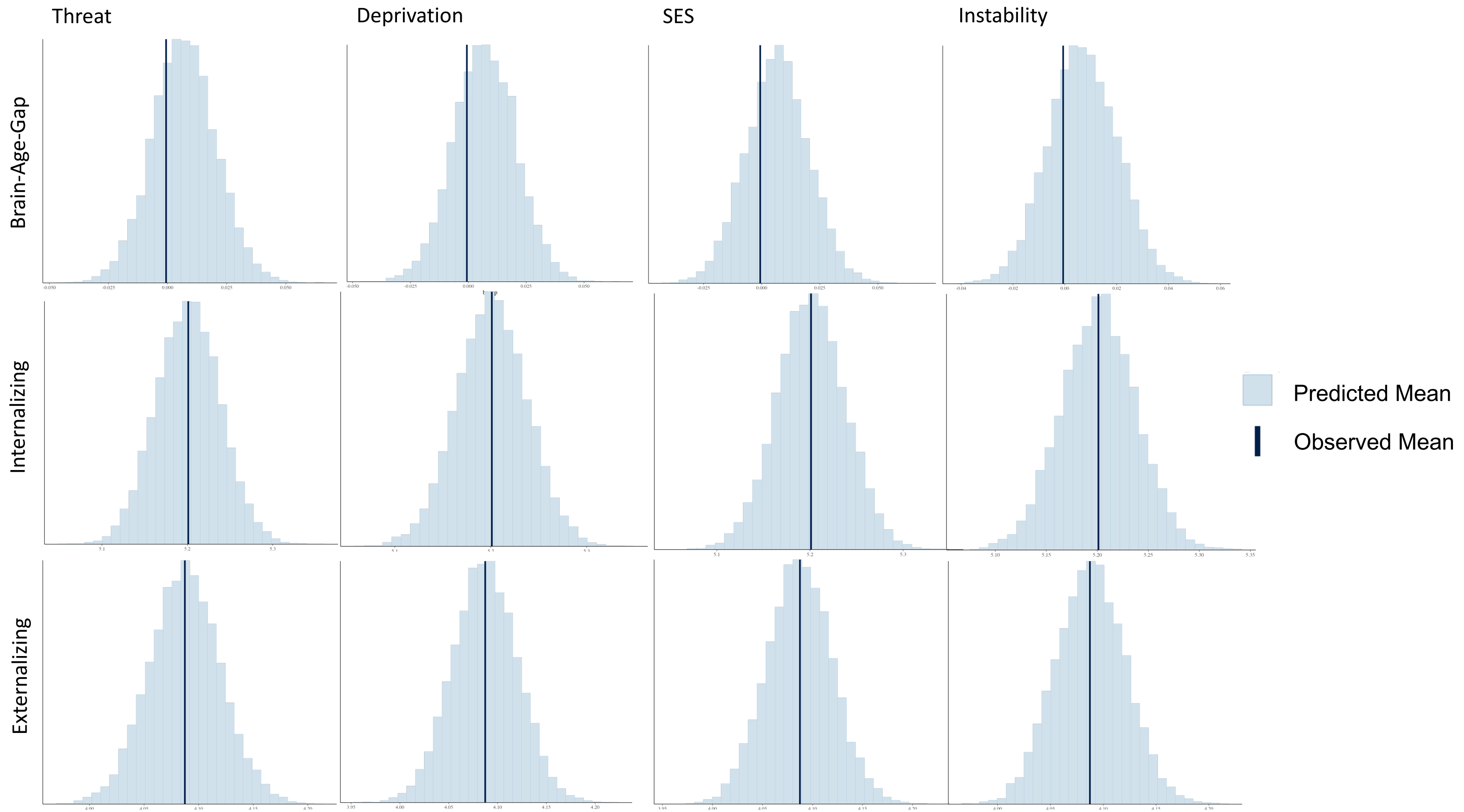

Figure S5

Proposed Conditional Effects Models

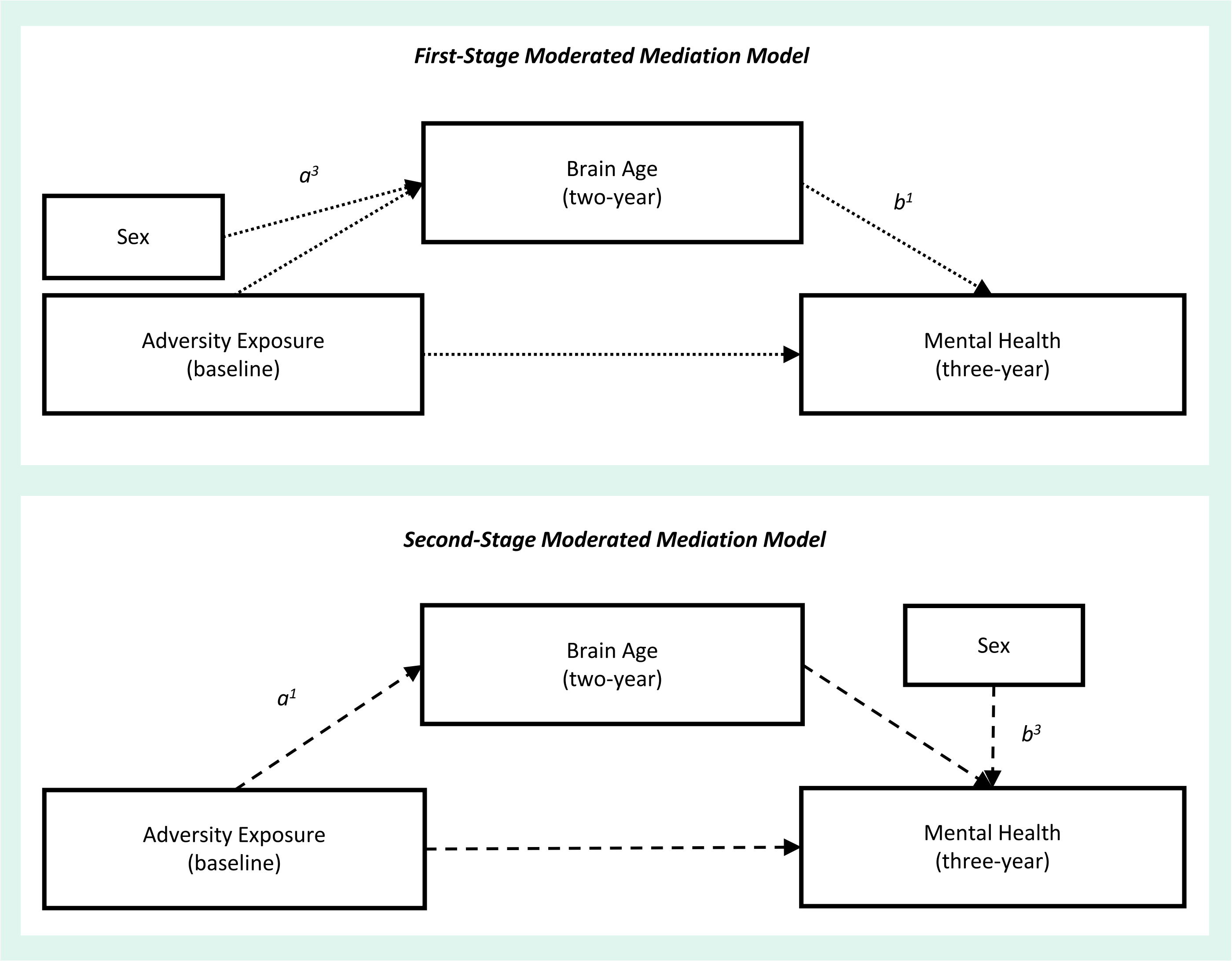

Results

Table S5

Descriptive Statistics and Statistical Comparison of Included and Excluded Participants

Variable	Included		Excluded		Comparison
	Freq/M(SD)	*%/Min-Max*	*Freq/M(SD)*	*%/Min-Max*	
n	7658		4207		
Sex					
Female	4111	54%	2080	49%	**χ^2(1) = 19.40, *p* <.001, *V* = 0.04[0.02, 1.00]**
Male	3547	46%	2127	51%	
COVID-19					**χ^2(1) = 267.05, *p* <.001, *V* = 0.15[0.13, 1.00]**
During	6410	84%	3960	94%	
Pre	1248	16%	247	6%	
Race/Ethnicity					**χ^2(4) = 105.15, *p* <.001, *V* = 0.09[0.08, 1.00]**
NH-White	4221	55%	1952	46%	
NH-Black	804	10%	443	11%	
Hispanic	1479	19%	930	22%	
NH-Asian	1010	13%	774	18%	
Other	144	2%	108	3%	
Age at 2-year follow up (years)	11.94 (0.65)	11-14	12.20 (0.67)	11-14	***t* = 20.57, *p* <.001, η^2 = .03[0.03, 1.00]**
Threat	0.12 (0.58)	-0.99-3.10	0.09 (0.57)	-1.00-3.10	*t* = 2.91, *p* = .004, η^2 = .001[0.00, 1.00]
Deprivation	-1.20 (0.60)	-2.50-1.10	-1.20 (0.61)	-2.50.1.30	*t* = 0.17, *p* = .865, η^2 < .001[0.00, 1.00]
SES	4.90 (2.40)	-4.40-9.30	5.00 (2.50)	-4.00-9.00	*t* = 1.07, *p* = .284, η^2 < .001[0.00, 1.00]
Instability	0.09 (0.51)	-0.85-2.30	0.07 (0.51)	-0.84-2.10	*t* = 2.62, *p* = .009, η^2= .001[0.00, 1.00]
Internalizing (3-year follow up)	5.20 (5.90)	0-44	5.00 (5.60)	0-49	*t* = -1.63, *p* = .103, η^2 < .001[0.00, 1.00]
Externalizing (3-year follow up)	4.10 (5.60)	0-48	3.80 (5.20)	0-49	*t* = -2.64, *p* = .008, η^2 = .001[0.00, 1.00]

Note. Freq, frequency; M, mean; SD, standard deviation; %, percentage of sex-specific sample; Min, minimum value; Max, maximum value; *n,* number of participants; NH, non-Hispanic; COVID-19, indicates whether three-year follow-up data was collected during or before the March 2020 pandemic. Statistically meaningful comparisons (*p* <.001) indicated in bold.

Figure S6

Mosaic and Violin Plots Comparing Included and Excluded Participants on Demographic Variables

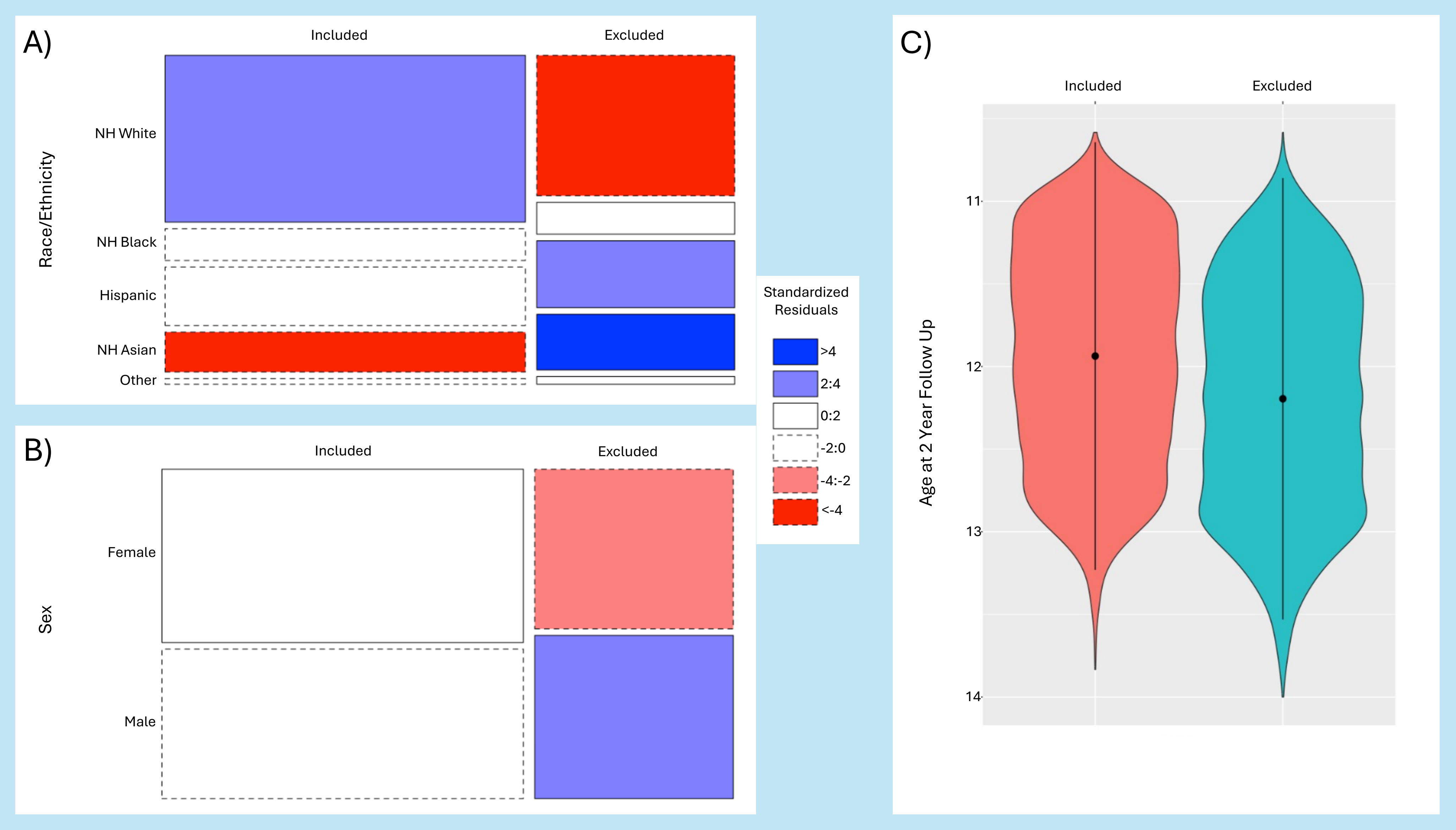

Note. Mosaic Plots: Blue = More likely, Red = Less likely

Table S6

Correlation Matrix of Predictor, Mediator, and Outcome Variables by Sex

		Males						
		Threat	Dep	SES	Instab	BAG	Internal	External
Females	Threat	-	0.25**	0.45**	0.71**	0.03	0.25**	0.27**
	Dep	0.29**	-	0.20**	0.20**	-0.01	0.10**	0.13**
	SES	0.45**	0.26**	-	0.43**	0.07**	0.06**	0.12**
	Instab	0.72**	0.26**	0.44**	-	0.04	0.27**	0.29**
	BAG	0.07**	0.05*	0.11**	0.05*	-	-0.01	0.02
	Internal	0.18**	0.06**	0.00	0.19**	0.03	-	0.60**
	External	0.25**	0.14**	0.10**	0.25**	0.04	0.55**	-

Note. Pearson's *r* correlations between adversity dimensions, brain-age-gap, and mental health problems displayed separately for sex assigned at birth. Females presented in bottom-left corner and males in the top-left corner. *p < .01; **p < .001. Dep, deprivation; SES, socioeconomic stress; Instab, household instability; BAG, brain-age-gap; Internal, internalizing problems; External, externalizing problems.

Table S7

Factor Loadings for Adversity Items in Four Factor Model

Factor	ABCD Item (Questionnaire Short Name)	Estimate	SE	*z*-value	*p*-value	Std.lv
threat =~						
	ksads_ptsd_raw_763_p (abcd_ptsd01)	1.000				0.857
	ksads_ptsd_raw_764_p (abcd_ptsd01)	0.990	0.026	38.330	0.000	0.848
	ksads_ptsd_raw_765_p (abcd_ptsd01)	1.032	0.026	39.980	0.000	0.884
	ksads_ptsd_raw_768_p (abcd_ptsd01)	0.990	0.025	38.871	0.000	0.848
	ksads_ptsd_raw_769_p (abcd_ptsd01)	0.842	0.028	30.056	0.000	0.721
	ksads_ptsd_raw_766_p (abcd_ptsd01)	0.972	0.030	32.743	0.000	0.832
	ksads_ptsd_raw_758_p (abcd_ptsd01)	1.136	0.034	33.377	0.000	0.973
	ksads_ptsd_raw_759_p (abcd_ptsd01)	1.082	0.027	39.405	0.000	0.927
	ksads_ptsd_raw_760_p (abcd_ptsd01)	0.945	0.023	41.024	0.000	0.809
	fes_youth_q3 (abcd_fes01)	0.456	0.030	15.438	0.000	0.390
	kbi_p_c_bully (dibf01)	0.526	0.028	18.861	0.000	0.451
	ksads_ptsd_raw_755_p (abcd_ptsd01)	0.464	0.033	14.038	0.000	0.397
	ksads_ptsd_raw_756_p (abcd_ptsd01)	0.642	0.031	20.899	0.000	0.550
	ksads_ptsd_raw_757_p (abcd_ptsd01)	0.579	0.031	18.537	0.000	0.496
	ksads_ptsd_raw_770_p (abcd_ptsd01)	0.456	0.023	19.591	0.000	0.391
deprivation =~						
	crpbi_parent1_y (crpbi01)	1.000				0.679
	crpbi_caregiver12_y (crpbi01)	0.565	0.018	30.602	0.000	0.384
	crpbi_parent2_y (crpbi01)	0.941	0.020	48.227	0.000	0.639
	crpbi_caregiver13_y (crpbi01)	0.599	0.019	30.937	0.000	0.407
	crpbi_parent3_y (crpbi01)	1.048	0.019	54.669	0.000	0.711
	crpbi_caregiver14_y (crpbi01)	0.619	0.019	32.337	0.000	0.420
	crpbi_parent4_y (crpbi01)	1.076	0.025	43.771	0.000	0.731
	crpbi_caregiver15_y (crpbi01)	0.595	0.022	27.256	0.000	0.404
	crpbi_parent5_y (crpbi01)	0.929	0.018	50.563	0.000	0.631
	crpbi_caregiver16_y (crpbi01)	0.536	0.019	28.425	0.000	0.364
	fes_youth_q1 (abcd_fes01)	-0.836	0.022	-38.258	0.000	-0.568
	fes_youth_q2 (abcd_fes01)	-0.601	0.021	-28.622	0.000	-0.408
	fes_youth_q4 (abcd_fes01)	-0.667	0.021	-31.699	0.000	-0.453
	fes_youth_q5 (abcd_fes01)	-0.729	0.022	-32.395	0.000	-0.495
	fes_youth_q7 (abcd_fes01)	-0.755	0.025	-29.756	0.000	-0.512
	fes_youth_q8 (abcd_fes01)	-0.555	0.022	-25.019	0.000	-0.377
	fes_youth_q9 (abcd_fes01)	-0.486	0.022	-22.389	0.000	-0.330
	parent_monitor_q1_y (pmq01)	0.655	0.021	31.826	0.000	0.445
	parent_monitor_q2_y (pmq01)	0.595	0.020	29.593	0.000	0.404
	parent_monitor_q3_y (pmq01)	0.499	0.020	25.054	0.000	0.339
	parent_monitor_q4_y (pmq01)	0.513	0.017	30.832	0.000	0.348
	parent_monitor_q5_y (pmq01)	0.521	0.019	28.009	0.000	0.354
ses =~						

	INR	1.000				2.582
	reshist_addr1_adi_perc (abcd_rhds01)	-6.528	0.174	-37.470	0.000	-16.853
	Mean Parent Education	0.639	0.018	36.425	0.000	1.649
instability =~						
	famhx_ss_parent_alc_p (abcd_fhxssp01)	1.000				0.686
	famhx_ss_parent_dg_p (abcd_fhxssp01)	0.975	0.021	45.415	0.000	0.668
	famhx_ss_parent_dprs_p (abcd_fhxssp01)	1.255	0.022	56.241	0.000	0.860
	famhx_ss_parent_ma_p (abcd_fhxssp01)	1.037	0.028	37.529	0.000	0.711
	famhx_ss_parent_nrv_p (abcd_fhxssp01)	0.926	0.024	38.271	0.000	0.635
	famhx_ss_parent_hspd_p (abcd_fhxssp01)	1.165	0.024	48.083	0.000	0.799
	famhx_ss_parent_scd_p (abcd_fhxssp01)	1.089	0.027	40.059	0.000	0.747
	famhx_ss_parent_trb_p (abcd_fhxssp01)	1.257	0.023	55.231	0.000	0.862
	Biological Parent Separation	0.679	0.030	22.370	0.000	0.465

Note. Estimate, value of the latent parameters; SE, standard error; Std.lv, parameter estimate with latent variables standardized; ses, socioeconomic stressors; INR, income-to-needs ratio (total family income (demo_comb_income_v2) divided by poverty threshold based on household size (demo_roster_v2)); Mean Parent Education (mean score across demo_prnt_ed_v2_l and demo_prtnr_ed_v2_l); Biological Parent Separation (positive endorsement of kbi_p_c_guard__[3-12] or demo_prim).

Table S8

Factor Loadings for Covariances in Four Factor Model

Covariances		Estimate	SE	z-value	p-value	Std.lv
crpbi_caregiver12_y ~~						
	crpbi_caregiver14_y	0.720	0.009	82.058	0.000	0.720
	crpbi_caregiver16_y	0.687	0.008	81.117	0.000	0.687
crpbi_caregiver14_y ~~						
	crpbi_caregiver16_y	0.670	0.009	75.849	0.000	0.670
crpbi_caregiver13_y ~~						
	crpbi_caregiver14_y	0.646	0.009	69.907	0.000	0.646
crpbi_caregiver12_y ~~						
	crpbi_caregiver13_y	0.646	0.009	72.676	0.000	0.646
crpbi_caregiver13_y ~~						
	crpbi_caregiver16_y	0.625	0.009	70.172	0.000	0.625
crpbi_caregiver14_y ~~						
	crpbi_caregiver15_y	0.728	0.010	75.197	0.000	0.728
crpbi_caregiver13_y ~~						
	crpbi_caregiver15_y	0.726	0.010	75.866	0.000	0.726
crpbi_caregiver12_y ~~						
	crpbi_caregiver15_y	0.727	0.009	78.735	0.000	0.727
crpbi_caregiver15_y ~~						
	crpbi_caregiver16_y	0.687	0.009	74.682	0.000	0.687
threat ~~						
	deprivation	-0.097	0.011	-9.214	0.000	-0.167
	ses	-0.650	0.039	-16.756	0.000	-0.294
	instability	0.294	0.013	22.066	0.000	0.501
deprivation ~~						
	ses	0.371	0.021	17.387	0.000	0.212
	instability	-0.080	0.007	-11.320	0.000	-0.172
ses ~~						
	instability	-0.587	0.023	-25.064	0.000	-0.332

Note. Estimate, value of the latent parameters; SE, standard error; Std.lv, parameter estimate with latent variables standardized.

Figure S7

Distribution of Brain Age Gap Values by Sex Assigned at Birth

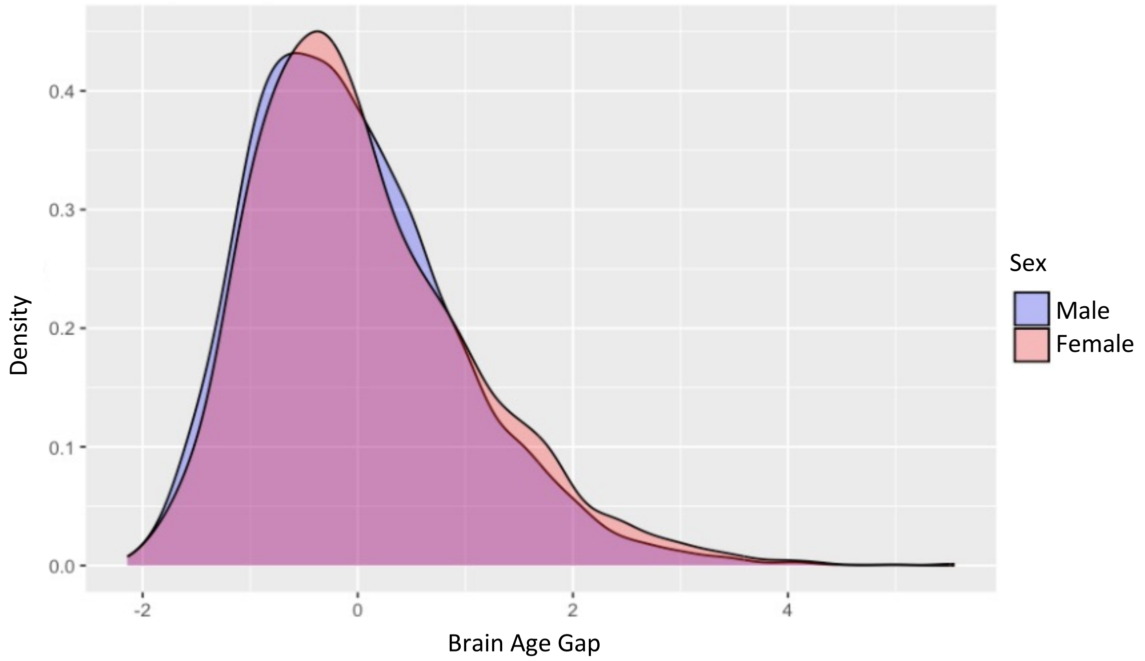

Figure S8

Examination of Age Bias in Brain Age Prediction

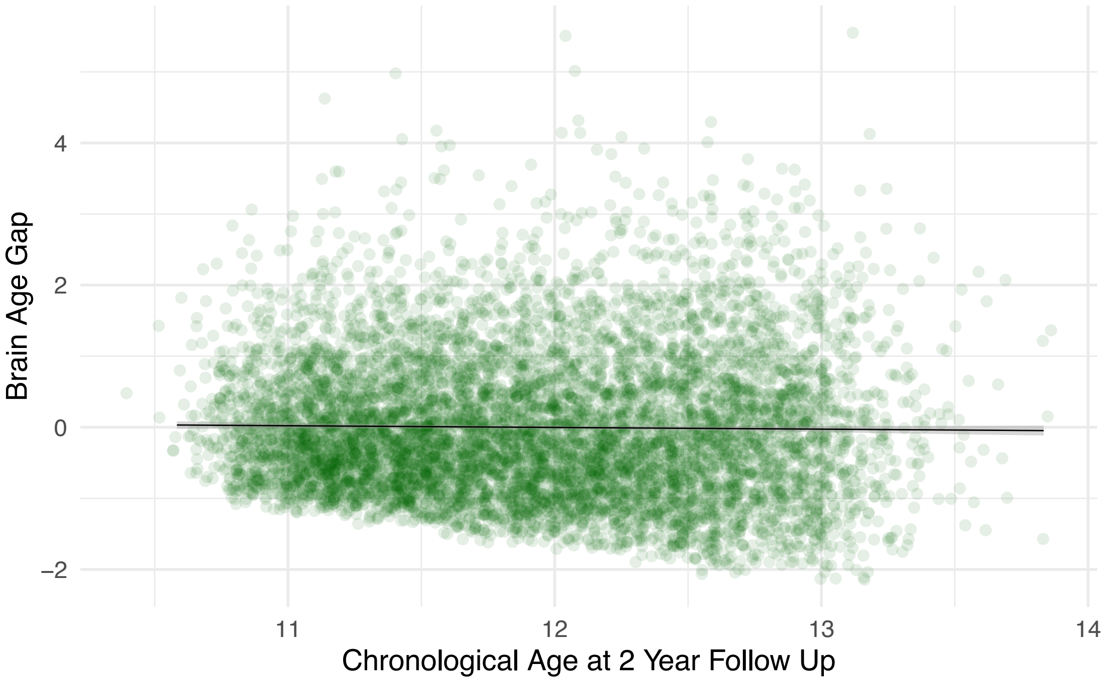

Table S9

Sensitivity Analysis: Bayesian Regression Posterior Estimates for Direct Pathways in Mediation Models

		Adversity - Brain Age Gap						Brain Age Gap - MH Problems						Adversity - MH Problems					
		Est	Err	95% L	95% U	99% L	99% U	Est	Err	95% L	95% U	99% L	99% U	Est	Err	95% L	95% U	99% L	99% U
Threat																			
Internalizing	*Baseline*	0.0066	0.0102	-0.0134	0.0267	-0.0192	0.0327	0.0208	0.0153	-0.0092	0.0502	-0.0174	0.0599	**0.0879**	0.0116	0.0651	0.1105	0.0583	0.1180
	Dimension	0.0045	0.0139	-0.0230	0.0318	-0.0316	0.0403	0.0146	0.0155	-0.0152	0.0448	-0.0242	0.0529	**0.1370**	0.0169	0.1038	0.1702	0.0944	0.1813
Externalizing	*Baseline*	0.0088	0.0104	-0.0114	0.0294	-0.0188	0.0355	**0.0554**	0.0177	0.0211	0.0900	0.0095	0.1017	**0.0952**	0.0130	0.0698	0.1201	0.0610	0.1285
	Dimension	0.0062	0.0139	-0.0214	0.0334	-0.0301	0.0416	**0.0498**	0.0175	0.0154	0.0845	0.0035	0.0943	**0.1047**	0.0188	0.0682	0.1416	0.0564	0.1544
Deprivation																			
Internalizing	*Baseline*	-0.0070	0.0094	-0.0254	0.0112	-0.0316	0.0177	0.0246	0.0150	-0.0048	0.0539	-0.0135	0.0642	**0.0336**	0.0102	0.0139	0.0539	0.0073	0.0599
	Dimension	-0.0090	0.0098	-0.0280	0.0104	-0.0335	0.0163	0.0171	0.0157	-0.0142	0.0470	-0.0239	0.0558	**0.0448**	0.0118	0.0222	0.0692	0.0145	0.0766
Externalizing	*Baseline*	-0.0063	0.0095	-0.0249	0.0122	-0.0304	0.0181	**0.0569**	0.0174	0.0222	0.0914	0.0123	0.1021	**0.0454**	0.0119	0.0220	0.0686	0.0149	0.0753
	Dimension	-0.0085	0.0097	-0.0277	0.0103	-0.0332	0.0168	**0.0507**	0.0175	0.0163	0.0848	0.0056	0.0959	**0.0732**	0.0131	0.0476	0.0994	0.0397	0.1078
SES																			
Internalizing	*Baseline*	0.0048	0.0114	-0.0173	0.0270	-0.0243	0.0336	0.0241	0.0153	-0.0058	0.0542	-0.0152	0.0644	-0.0058*	0.0139	-0.0333	0.0217	-0.0421	0.0298
	Dimension	0.0020	0.0125	-0.0222	0.0264	-0.0295	0.0337	0.0135	0.0153	-0.0166	0.0436	-0.0266	0.0526	**-0.0781**	0.0177	-0.1134	-0.0430	-0.1246	-0.0322
Externalizing	*Baseline*	0.0048	0.0114	-0.0177	0.0273	-0.0242	0.0342	**0.0571**	0.0174	0.0229	0.0909	0.0125	0.1022	**0.0329**	0.0167	0.0008	0.0659	-0.0088	0.0779
	Dimension	0.0012	0.0127	-0.0236	0.0265	-0.0309	0.0337	**0.0504**	0.0173	0.0155	0.0842	0.0036	0.0938	-0.0233	0.0219	-0.0655	0.0198	-0.0793	0.0340
Instability																			
Internalizing	*Baseline*	0.0085	0.0103	-0.0117	0.0287	-0.0179	0.0352	0.0205	0.0148	-0.0087	0.0498	-0.0170	0.0590	**0.1071**	0.0118	0.0837	0.1304	0.0759	0.1375
	Dimension	0.0088	0.0139	-0.0178	0.0358	-0.0272	0.0459	0.0138	0.0153	-0.0162	0.0443	-0.0256	0.0529	**0.1703**	0.0183	0.1341	0.2064	0.1229	0.2171
Externalizing	*Baseline*	0.0098	0.0102	-0.0100	0.0300	-0.0157	0.0355	**0.0444**	0.0207	0.0050	0.0825	-0.0050	0.0942	**0.1431**	0.0139	0.1163	0.1704	0.1080	0.1799
	Dimension	0.0087	0.0140	-0.0189	0.0357	-0.0270	0.0447	**0.0496**	0.0174	0.0156	0.0835	0.0052	0.0945	**0.2394**	0.0213	0.1978	0.2813	0.1846	0.2930

Note. Bayesian mixed-effects regression models were used to estimate associations between adversity dimensions (threat, deprivation, socio-economic stressors [SES], and household instability), brain-age-gap, and mental health problems [MH Problems] (internalizing and externalizing). Each row of the table

represents a separate model. Est, mean of the posterior distribution; Err, standard deviation of the posterior distribution; 95% L, lower-bound of credibility intervals calculated at 95% quantiles of the posterior distribution; 95% U, upper-bound of 95% credibility intervals; 99% L, lower-bound of credibility intervals calculated at 99% quantiles of the posterior distribution; 99% U, upper-bound of 99% credibility intervals; Baseline, baseline mental health problems included as a covariate; Dimension, the other adversity latent factor scores included as covariates; * indicates change from primary models.

Table S10

Bayesian Posterior Estimates of the Indirect Effect and Proportion Mediated

		Indirect Effect (*a*b*)					Proportion Mediated				
		Est	95% CI L	95% CI U	99% CI L	99% CI U	Est	95% CI L	95% CI U	99% CI L	99% CI U
Threat											
Internalizing	*Primary*	0.0001	-0.0003	0.0008	-0.0005	0.0011	0.0006	-0.0012	0.0034	-0.0020	0.0047
	Baseline	0.0001	-0.0003	0.0008	-0.0005	0.0012	0.0016	-0.0039	0.0095	-0.0065	0.0142
	Dimension	0.0001	-0.0005	0.0008	-0.0008	0.0013	0.0006	-0.0037	0.0061	-0.0063	0.0098
Externalizing	*Primary*	0.0006	-0.0004	0.0020	-0.0008	0.0026	0.0025	-0.0017	0.0080	-0.0032	0.0105
	Baseline	0.0005	-0.0006	0.0019	-0.0011	0.0025	0.0053	-0.0068	0.0205	-0.0115	0.0277
	Dimension	0.0003	-0.0011	0.0020	-0.0017	0.0027	0.0032	-0.0113	0.0196	-0.0182	0.0290
Deprivation											
Internalizing	*Primary*	-0.0001	-0.0008	0.0004	-0.0012	0.0007	-0.0017	-0.0099	0.0043	-0.0142	0.0080
	Baseline	-0.0002	-0.0009	0.0003	-0.0012	0.0006	-0.0055	-0.0327	0.0110	-0.0550	0.0201
	Dimension	-0.0002	-0.0008	0.0003	-0.0011	0.0005	-0.0038	-0.0215	0.0064	-0.0345	0.0119
Externalizing	*Primary*	-0.0003	-0.0015	0.0009	-0.0020	0.0013	-0.0025	-0.0135	0.0076	-0.0177	0.0113
	Baseline	-0.0004	-0.0016	0.0007	-0.0020	0.0012	-0.0086	-0.0431	0.0176	-0.0657	0.0320
	Dimension	-0.0004	-0.0016	0.0005	-0.0021	0.0010	-0.0062	-0.0245	0.0077	-0.0339	0.0137
SES											
Internalizing	*Primary*	0.0002	-0.0005	0.0010	-0.0008	0.0014	0.0020	-0.0064	0.0138	-0.0112	0.0209
	Baseline	0.0001	-0.0005	0.0009	-0.0008	0.0012	0.0246	-0.2665	0.2266	-1.1303	1.3527
	Dimension	0.0000	-0.0005	0.0006	-0.0008	0.0010	-0.0003	-0.0086	0.0073	-0.0142	0.0132
Externalizing	*Primary*	0.0004	-0.0010	0.0019	-0.0015	0.0026	0.0024	-0.0061	0.0122	-0.0095	0.0166
	Baseline	0.0003	-0.0011	0.0018	-0.0017	0.0023	0.0005	-0.0599	0.1082	-0.3476	0.4042
	Dimension	0.0001	-0.0013	0.0015	-0.0019	0.0021	0.0100	-0.1981	0.2176	-0.8067	1.0015
Instability											
Internalizing	*Primary*	0.0002	-0.0003	0.0009	-0.0005	0.0013	0.0008	-0.0010	0.0036	-0.0019	0.0051
	Baseline	0.0002	-0.0003	0.0009	-0.0006	0.0013	0.0016	-0.0029	0.0085	-0.0052	0.0121
	Dimension	0.0001	-0.0004	0.0009	-0.0008	0.0013	0.0007	-0.0026	0.0054	-0.0044	0.0080
Externalizing	*Primary*	0.0006	-0.0004	0.0019	-0.0008	0.0025	0.0019	-0.0012	0.0059	-0.0026	0.0079
	Baseline	0.0004	-0.0005	0.0017	-0.0009	0.0023	0.0031	-0.0033	0.0123	-0.0062	0.0165
	Dimension	0.0004	-0.0010	0.0021	-0.0016	0.0028	0.0018	-0.0042	0.0087	-0.0070	0.0119

Note. Bayesian mixed-effects regression models were used to estimate the indirect effect of brain-age-gap on associations between adversity dimensions (threat, deprivation, socioeconomic stress, and household instability) and mental health problems (internalizing and externalizing). Est, Estimate; CI L, lower bound credibility interval; CI U, upper bound credibility interval (CIs calculated at 95 and 99% quantiles of the posterior distributions); MH, mental health; Primary, primary analysis model; Baseline, baseline mental health problems included as a covariate; Dimension, the other adversity latent factor scores included as covariates.

Table S11

Bayesian Regression Mediation Models Separate by Sex Assigned at Birth

		Adversity - Brain Age Gap (path *a*)				Brain Age Gap - MH Problems (path *b*)				Adversity - MH Problems (path c')				Indirect Effect (*a***b*)		
		Est	*Err*	*95% CI*		*Est*	*Err*	*95% CI*		*Est*	*Err*	*95% CI*		*Est*	*95% CI*	
Internalizing																
Threat	F	0.0219	0.0145	-0.0066	0.0500	0.0164	0.0236	-0.0294	0.0630	**0.2033**	0.0192	0.1659	0.2404	0.0004	-0.0008	0.0020
	M	-0.0012	0.0130	-0.0269	0.0245	0.0082	0.0237	-0.0386	0.0547	**0.2809**	0.0184	0.2453	0.3167	0.0000	-0.0007	0.0007
Deprivation	F	0.0131	0.0138	-0.0140	0.0400	0.0288	0.0232	-0.0171	0.0740	**0.0640**	0.0167	0.0311	0.0964	0.0004	-0.0006	0.0019
	M	**-0.0263**	0.0129	-0.0523	-0.0011	0.0119	0.0234	-0.0344	0.0573	**0.1162**	0.0168	0.0832	0.1493	-0.0003	-0.0019	0.0011
SES	F	0.0177	0.0164	-0.0139	0.0502	0.0288	0.0232	-0.0171	0.0740	0.0328	0.0237	-0.0143	0.0789	0.0005	-0.0006	0.0022
	M	-0.0038	0.0151	-0.0330	0.0256	0.0119	0.0234	-0.0344	0.0573	**0.1198**	0.0228	0.0758	0.1642	0.0000	-0.0010	0.0008
Instability	F	0.0189	0.0147	-0.0095	0.0479	0.0195	0.0235	-0.0266	0.0657	**0.2162**	0.0196	0.1772	0.2546	0.0004	-0.0007	0.0018
	M	0.0045	0.0133	-0.0213	0.0303	0.0054	0.0235	-0.0414	0.0507	**0.3154**	0.0196	0.2771	0.3534	0.0000	-0.0007	0.0008
Externalizing																
Threat	F	0.0247	0.0145	-0.0042	0.0532	**0.1252**	0.0296	0.0662	0.1827	**0.2873**	0.0229	0.2419	0.3330	0.0031	-0.0005	0.0075
	M	-0.0027	0.0130	-0.0278	0.0228	-0.0136	0.0250	-0.0633	0.0352	**0.3010**	0.0205	0.2606	0.3417	0.0001	-0.0007	0.0010
Deprivation	F	0.0175	0.0138	-0.0094	0.0447	**0.1448**	0.0296	0.0862	0.2036	**0.1078**	0.0210	0.0671	0.1495	0.0026	-0.0013	0.0072
	M	**-0.0265**	0.0128	-0.0510	-0.0012	-0.0104	0.0250	-0.0583	0.0395	**0.1656**	0.0186	0.1299	0.2019	0.0003	-0.0011	0.0020
SES	F	0.0168	0.0171	-0.0135	0.0536	**0.1465**	0.0373	0.0782	0.2180	**0.1523**	0.0317	0.1003	0.2164	0.0025	-0.0019	0.0099
	M	-0.0033	0.0153	-0.0332	0.0269	-0.0054	0.0251	-0.0545	0.0436	**0.1687**	0.0279	0.1141	0.2233	0.0000	-0.0008	0.0009
Instability	F	0.0197	0.0143	-0.0087	0.0476	**0.1211**	0.0293	0.0637	0.1789	**0.3061**	0.0241	0.2581	0.3534	0.0024	-0.0010	0.0065
	M	0.0042	0.0134	-0.0224	0.0301	-0.0183	0.0248	-0.0672	0.0300	**0.3729**	0.0224	0.3285	0.4168	-0.0001	-0.0011	0.0008

Note. Primary mediation models run separately for males and females. Est, Estimate; 95% CI, lower and upper bound of 95% quantile credibility intervals.

References

Bürkner, P.-C. (2017). brms: An R Package for Bayesian Multilevel Models Using Stan. *Journal of Statistical Software*, *80*(1). https://doi.org/10.18637/jss.v080.i01

Gelman, A., Simpson, D., & Betancourt, M. (2017). The Prior Can Often Only Be Understood in the Context of the Likelihood. *Entropy*, *19*(10). https://doi.org/10.3390/e19100555

Hagler, D. J., Jr., Hatton, S., Cornejo, M. D., Makowski, C., Fair, D. A., Dick, A. S., Sutherland, M. T., Casey, B. J., Barch, D. M., Harms, M. P., Watts, R., Bjork, J. M., Garavan, H. P., Hilmer, L., Pung, C. J., Sicat, C. S., Kuperman, J., Bartsch, H., Xue, F., . . . Dale, A. M. (2019). Image processing and analysis methods for the Adolescent Brain Cognitive Development Study. *NeuroImage*, *202*, 116091. https://doi.org/10.1016/j.neuroimage.2019.116091

Leonardsen, E. H., Peng, H., Kaufmann, T., Agartz, I., Andreassen, O. A., Celius, E. G., Espeseth, T., Harbo, H. F., Hogestol, E. A., Lange, A. M., Marquand, A. F., Vidal-Pineiro, D., Roe, J. M., Selbaek, G., Sorensen, O., Smith, S. M., Westlye, L. T., Wolfers, T., & Wang, Y. (2022). Deep neural networks learn general and clinically relevant representations of the ageing brain. *NeuroImage*, *256*, 119210. https://doi.org/10.1016/j.neuroimage.2022.119210

R Core Team. (2021). *R: A language and environment for statistical computing*. In R Foundation for Statistical Computing. https://www.R-project.org/

RStudio Team. (2020). *RStudio: Integrated Development for R*. In RStudio, PBC. http://www.rstudio.com/.

Van Buuren, S. (2012). *Flexible Imputation of Missing Data* (2nd ed.). CRC Press. https://stefvanbuuren.name/fimd/

Van Buuren, S., & Groothuis-Oudshoorn, K. (2011). mice: Multivariate Imputation by Chained Equations in R. *Journal of Statistical Software*, *45*(3). https://doi.org/10.18637/jss.v045.i03

Vehtari, A. (2024, *Prior Choice Recommendations*. Retrieved 7 May 2024 from https://github.com/stan-dev/stan/wiki/Prior-Choice-Recommendations

Vehtari, A., Gelman, A., Simpson, D., Carpenter, B., & Bürkner, P.-C. (2021). Rank-Normalization, Folding, and Localization: An Improved Rˆ for Assessing Convergence of MCMC (with Discussion). *Bayesian Analysis*, *16*(2). https://doi.org/10.1214/20-ba1221

Yuan, Y., & MacKinnon, D. P. (2009). Bayesian mediation analysis. *Psychological Methods*, *14*(4), 301-322. https://doi.org/10.1037/a0016972
